# Epigenetics in rare neurological diseases

**DOI:** 10.3389/fcell.2024.1413248

**Published:** 2024-07-23

**Authors:** Chris-Tiann Roberts, Khatereh Saei Arezoumand, Ashraf Kadar Shahib, James R. Davie, Mojgan Rastegar

**Affiliations:** Department of Biochemistry and Medical Genetics, Max Rady College of Medicine, Rady Faculty of Health Sciences, University of Manitoba, Winnipeg, MB, Canada

**Keywords:** Rett Syndrome, Prader-Willi Syndrome, Angelman Syndrome, rare neurological diseases, MeCP2, DNA methylation, histone modifications, epigenetics

## Abstract

Rare neurological diseases include a vast group of heterogenous syndromes with primary impairment(s) in the peripheral and/or central nervous systems. Such rare disorders may have overlapping phenotypes, despite their distinct genetic etiology. One unique aspect of rare neurological diseases is their potential common association with altered epigenetic mechanisms. Epigenetic mechanisms include regulatory processes that control gene expression and cellular phenotype without changing the composition of the corresponding DNA sequences. Epigenetic factors include three types of proteins, the “readers, writers, and erasers” of DNA and DNA-bound proteins. Thus, epigenetic impairments of many neurological diseases may contribute to their pathology and manifested phenotypes. Here, we aim to provide a comprehensive review on the general etiology of selected rare neurological diseases, that include Rett Syndrome, Prader-Willi Syndrome, Rubinstein-Taybi Syndrome, Huntington’s disease, and Angelman syndrome, with respect to their associated aberrant epigenetic mechanisms.

## 1 Introduction

**FIGURE 1:**
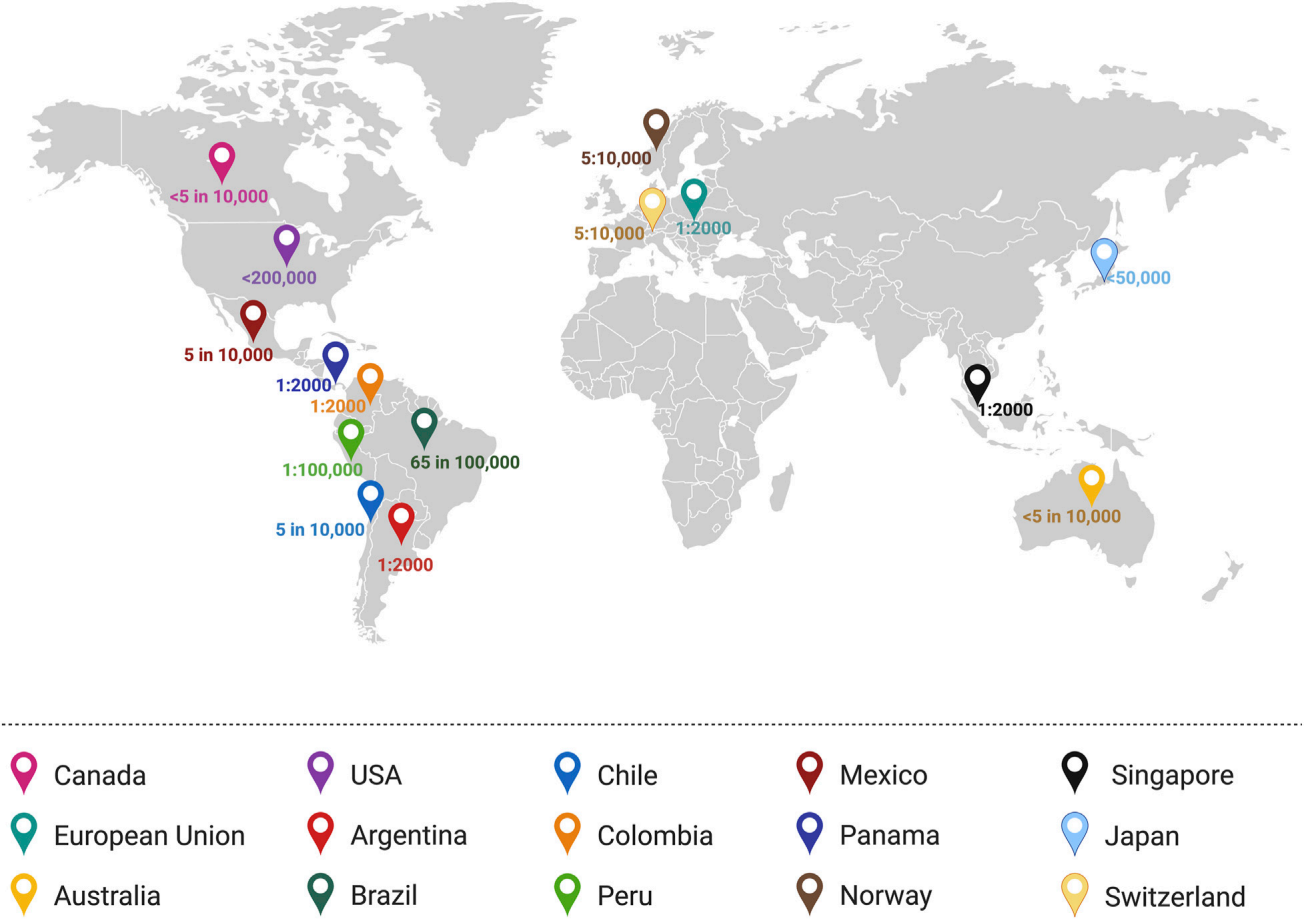
Worldwide definitions of “rare disease” based on the prevalence or incidence. Several countries define a rare disease as a disease which has an incidence of 1:2000 (Panama, Colombia, Argentina, Singapore, and the European Union). Meanwhile, other countries define rare diseases as fewer than 5 in 10,000 people (Canada and Australia) or other definitions as in the case of the United States (<200,000 people), Brazil (65 in 100,000 people), and Mexico (5 in 10,000 people) (Schouten, 2020). Illustration created in BioRender.com.

A rare disease would affect a small fraction of the population and in most cases, it is associated with a known genetic cause or a genetic component. The affected individuals may exhibit diverse symptoms with chronic and/or life-threatening impact (Haendel et al., 2020). While there is no uniquely standard definition for a “rare disease,” the term commonly reflects the prevalence of the disease within the general population. Accordingly, the affected proportion who are diagnosed with such diseases, as well as the frequency of these incidences, and the number of diagnoses per year, may vary by country (Figure 1). Further, diseases may be considered as “rare” in certain demographic groups or regions (Haendel et al., 2020). Regardless of the country-, region- or demographic-specific definition of a rare disease, the World Health Organization (WHO) uses the International Classification of Diseases (ICD) as a record of global health conditions (WHO, 2019). Using the latest WHO revision of the ICD, ICD-11, diseases may be referred to their ICD- 11 number (WHO, 2019). Thus, despite the lack of a global consensus on the definition of rare diseases, WHO ICD provides a common means by which, rare diseases could be referred (WHO, 2019).

There are an estimated 7,000 rare diseases, which impact a variety of organs within the body (Haendel et al., 2020). Rare diseases are further complicated by their genetic and phenotypic heterogeneity, which may lead to mis-diagnosis (Pogue et al., 2018). However, technological advancements have aided in the genetic diagnosis of rare diseases and development of therapeutic strategies for treatment of symptoms. Of the rare diseases known thus far, rare neurological diseases include a heterogeneous assortment of disorders affecting the peripheral nervous system (PNS) and/or central nervous system (CNS). Neurological diseases refer to a broad spectrum of pathologies that range from neurodegenerative diseases to neuroinflammatory and neurodevelopmental disorders (Borsook, 2012). Despite the vast variety of pathologies, a recurring molecular event that could drive rare neurological diseases is impaired epigenetic mechanisms. The regulatory role of epigenetics has been implicated in the development of the nervous system, diversification of neural cell types and formation of synaptic and neural networks (Salinas et al., 2020). It is well-accepted that aberrant epigenetic mechanisms contribute to the pathology of rare neurological diseases. Indeed, in some cases, the pathology of rare neurological diseases is related to impaired function of epigenetic factors, mutations in genes encoding epigenetic factors, and/or pathways regulated by epigenetics (Qureshi and Mehler, 2018). Here, we will discuss the role of certain epigenetic factors in disease mechanism and pathology of rare neurological diseases including Rett Syndrome, Prader Willi Syndrome, Angelman Syndrome, Rubinstein-Taybi Syndrome, and Huntington’s disease.

## 2 Rett Syndrome

### 2.1 Etiology and clinical symptoms

**FIGURE 2:**
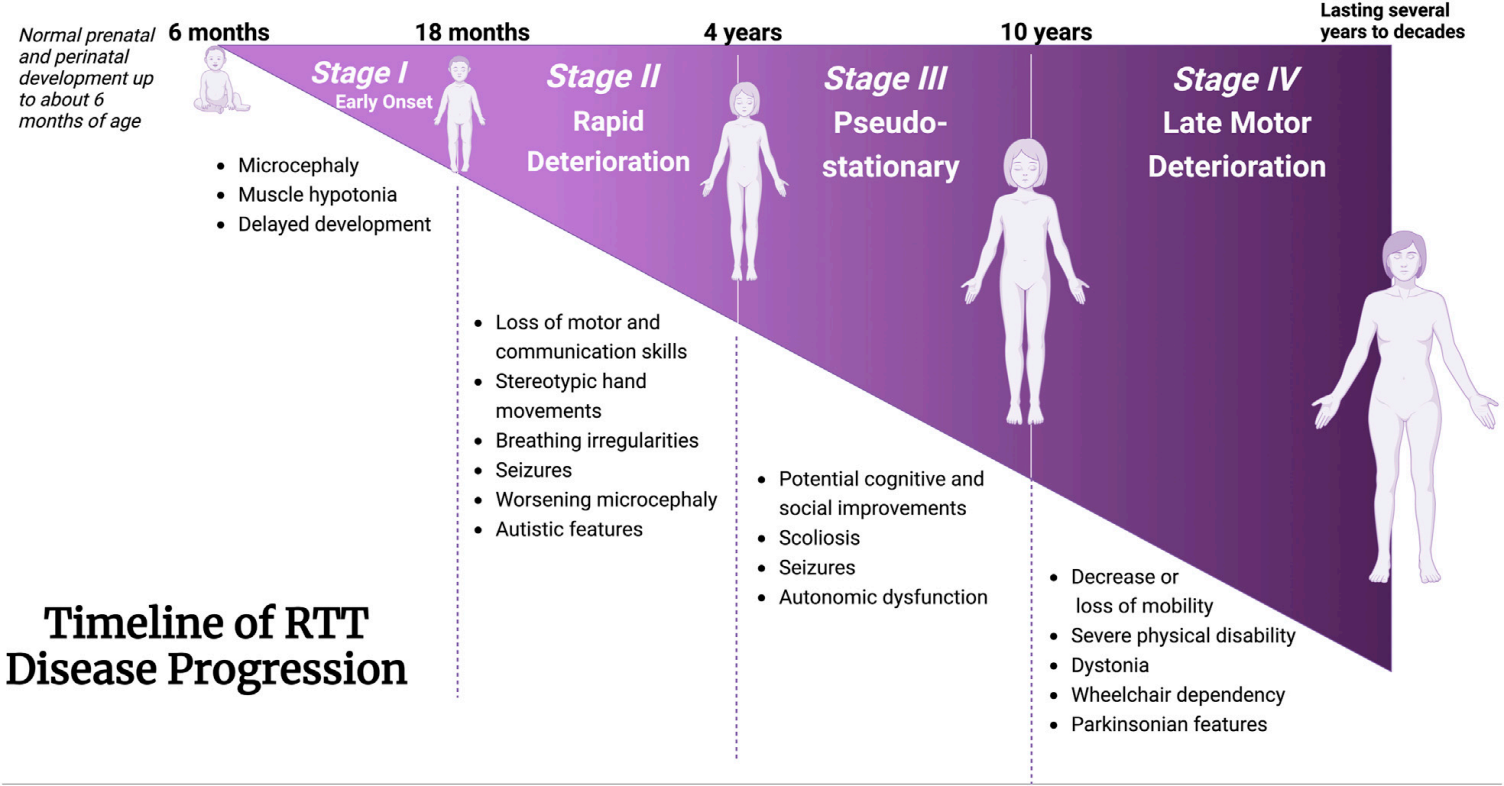
Timeline of clinical manifestation of Rett Syndrome. Characteristic features coinciding with disease progression from Stages I through IV are described. Stage I: Early onset; Stage II: Rapid Deterioration; Stage 3: Pseudo-stationary; Stage 4: Late motor deterioration. Schematic created using BioRender. Figure is adapted and information are extracted from Vashi and Justice (2019), and Kyle et al. (2018) (Kyle et al., 2018; Vashi and Justice, 2019).

Rett syndrome (RTT, OMIM 312750) is a severe X-linked and rare neurodevelopmental disorder with a frequency of 7.1 per 100,000 females (Petriti et al., 2023). After an apparently normal developmental period of 6–18 months of age, disease-associated symptoms develop through multiple steps (Figure 2), including stagnation, rapid regression, plateau/pseudo-stationary, and late motor deterioration (Hagberg, 2002; Kyle et al., 2018; Vashi and Justice, 2019). During the first stage of the disease (stagnation), RTT patients show developmental regression in their movement and speech abilities with reduced awareness. This stage is frequently ignored in RTT diagnosis, as the parents and physicians may not detect these slight and minor changes. Throughout the rapid regression stage, RTT patients also experience loss of control in their hand movement and ability to speak, in parallel to motor and breathing abnormalities (Vashi and Justice, 2019). Subsequently, development of autistic-like characteristics such as social avoidance, and seizures are exhibited in RTT patients. These conditions would result in the plateau stage that is characterized by motor challenges and seizures. Finally, RTT patients progress into the late motor descent stage, associated with strict physical disability and wheelchair-bounding conditions (Kyle et al., 2018). In addition to these specific disease stages, some patients may experience digestive struggles (Motil et al., 2012), reduced bone density (Shapiro et al., 2010), urological dysfunction (Ward et al., 2016), scoliosis (Menachem et al., 2023) osteoporosis (Haas et al., 1997), and sleep distresses (Young et al., 2007).

RTT patients have enhanced frequency of sudden mortality and may die *due to* breathing and cardiac abnormalities (Acampa and Guideri, 2006; Laurvick et al., 2006; Ramirez et al., 2013). Using gene screening methodologies, mutation of the methyl-CpG-binding protein 2 (*MECP2*) gene was recognized as the underlying cause of most RTT cases (Amir et al., 1999). Accordingly, *de novo* mutation in the *MECP2* gene was established to be the main causative genetic basis in over 95% of typical RTT cases (Bienvenu et al., 2000). Even though approximately 600 identified mutations leading to RTT have been detected in *MECP2*, genetic mutations that lead to 8 missense and nonsense mutations in the MeCP2 protein (R106W, R133C, T158M, R168X, R255X, R270X, R294X, and R306C) comprise about 70% of all mutations in RTT (Neul et al., 2008). An additional 15% of *MECP2* gene mutations happen *due to* large deletions within the *MECP2* gene. Of note, *MECP2-*associated genetic mutations have also been linked to intellectual impairments (Bianciardi et al., 2016) and autism (Xi et al., 2011).

### 2.2 The *MECP2* gene structure and MeCP2 protein function

**FIGURE 3:**
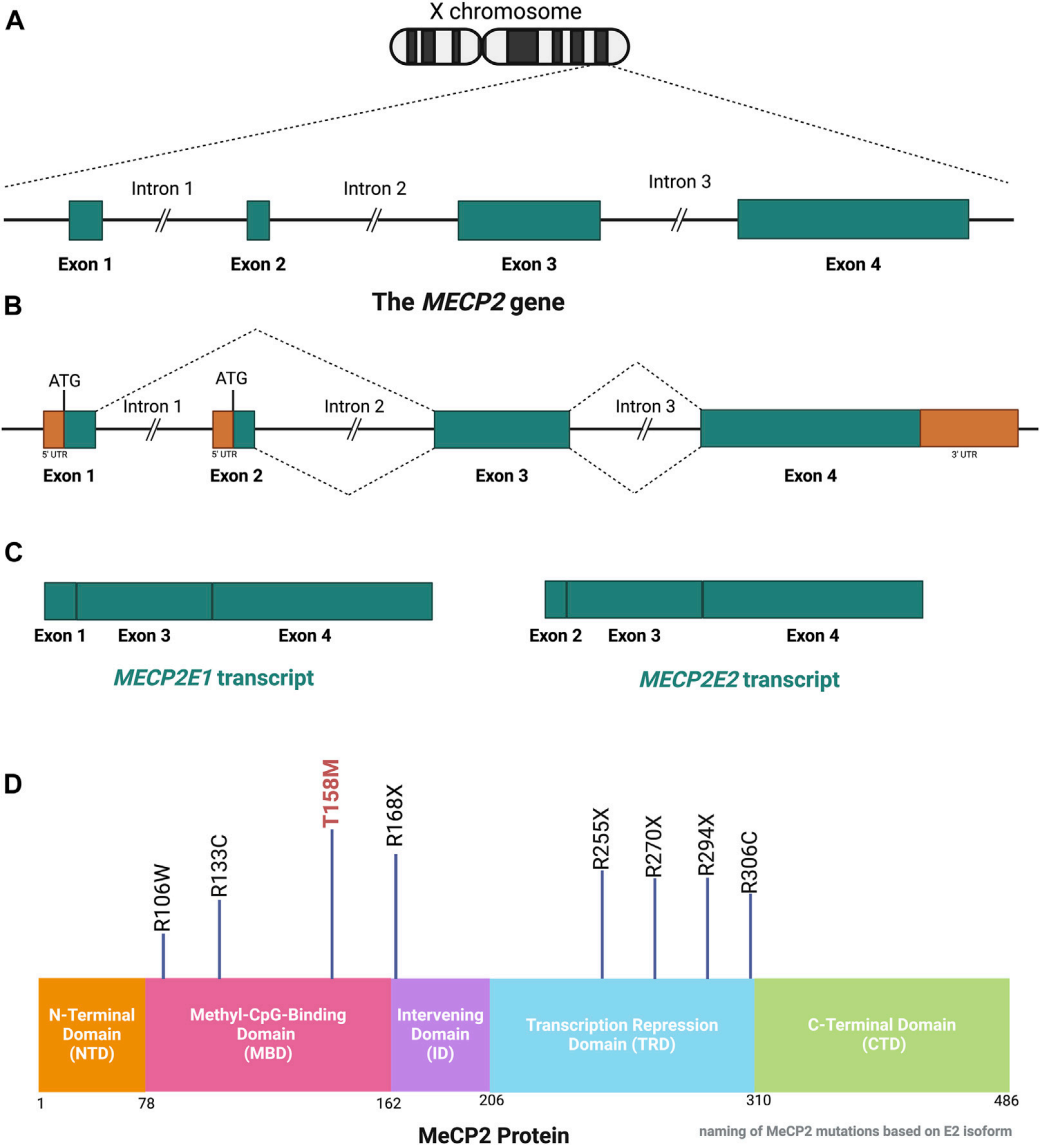
Schematic of the *MECP2* Gene and MeCP2 Protein Structures. **(A)** The *MECP2* gene is located on the X-chromosome. **(B, C)** Alternative splicing of the *MECP2* gene generates the two isoform transcripts, namely, *MECP2E1* and *MECP2E2*. The ATG is the translation start site, which is indicated for each isoform. **(D)** The MeCP2 protein contains 5 domains, each with specific functions. Eight mutations which account for over 70% of RTT cases are shown with their positions in each domain referenced by the number of amino acids in the MeCP2E2 isoform. The T158M mutation is regarded as the most common RTT-causing mutation. This figure has been adapted from Kyle et al., 2018; Liyanage and Rastegar 2014; Pejhan and Rastegar 2021. *MECP2*: *Methyl-CpG Binding Protein 2*. Coding sequences, non-coding sequences, and UTR: Untranslated Region. CTD: C-Terminal Domain, ID: Intervening Domain, MBD: Methyl-CpG-Binding Domain*,* NTD: N-terminal Domain, TRD: Transcriptional Repression Domain. Illustration created in BioRender.com.

The MeCP2 protein is a DNA methyl-binding protein with various levels of expression in different tissues, being abundantly expressed within neurons (Lewis et al., 1992). The *MECP2* gene (Figure 3) is located on the X-chromosome (Xq28) and is around 76 kb (Quaderi et al., 1994), including the transcription start site (TSS), four exons, three introns, and multiple polyadenylation (Poly A) sites, giving rise to the formation of two well-known protein isoforms (MeCP2E1 and MeCP2E2) by alternative splicing (Kriaucionis and Bird, 2004; Olson et al., 2014). Exons 1, 3, and 4 encode MeCP2E1 as the dominant isoform in the brain with relatively uniform levels in the cortex, hippocampus, thalamus, olfactory bulb, striatum, cerebellum, and brainstem (Kriaucionis and Bird, 2004; Mnatzakanian et al., 2004; Zachariah et al., 2012; Olson et al., 2014). Exons 2, 3, and 4 encode MeCP2E2, which is expressed at higher levels than MeCP2E1 in the placenta and liver, but in lower levels in both the murine and human brain (Mnatzakanian et al., 2004; Olson et al., 2014; Pejhan et al., 2020). The translation start site (ATG) within the exon 1 and exon 2 are used for the formation of MeCP2E1 and MeCP2E2 proteins that differ only in their N-termini with twenty-one unique amino acids in MeCP2E1, and nine specific amino acids to MeCP2E2 (Kriaucionis and Bird, 2004; Mnatzakanian et al., 2004; Liyanage and Rastegar, 2014). Additionally, studies have shown that MeCP2E1 is evenly expressed across diverse brain regions, while MeCP2E2 displays different levels of expression across mouse brain regions (Olson et al., 2014). In addition to these two commonly studied MeCP2 isoforms, additional coding, and non-coding *MECP2* transcripts have been reported by *in silico* studies (Shevkoplyas et al., 2022). Expression of *Mecp2* isoforms during differentiation of brain-derived neural stem cells is reciprocal (Liyanage et al., 2013). Also, a recent report of the transcriptional inhibitory feedback of MeCP2E1 and MeCP2E2 at the *Mecp2* promoter activity is suggestive of auto-regulatory mechanisms between the two isoforms in brain cells (Lockman et al., 2024). At the protein level, MeCP2E2 overexpression leads to degradation of MeCP2E1 protein through a proteosome-mediated pathway (Buist et al., 2022). In support of MeCP2-specific auto-regulation, MeCP2E1 deficiency in mice leads to elevation of MeCP2E2 protein levels in the brain, also suggesting an inhibitory effect of MeCP2E1 on MeCP2E2 (Yasui et al., 2014).

MeCP2 is an epigenetic regulator of gene transcription, which mediates its role *via* binding to the chromatin and DNA (both methylated and unmethylated) in cooperation with other proteins and complexes (Nan et al., 1997; Chandler et al., 1999; Hansen et al., 2010; Bin Akhtar et al., 2022). Initially, MeCP2 was defined as a suppressor of methylated DNA transcription *via* association with a co-repressor complex including mSin3A, a transcriptional repressor, and histone deacetylases, which can lead to chromatin compaction and gene silencing (Jones et al., 1998). Contrary to the initial discovery, later findings showed that MeCP2 can be an activator of gene expression *via* connection with CREB (cAMP response element-binding protein), which is an activating factor (Chahrour et al., 2008). The increased expression of MeCP2 is associated with neuronal maturation (Kishi and Macklis, 2004). The MeCP2 protein has multiple functional domains. MeCP2 protein also includes three AT-hook domains, which are located in the Transcription Repression Domain (TRD), C-terminal Domain (CTD), and Intervening Domain (ID) to facilitate the AT-rich DNA binding ability of MeCP2 (Baker et al., 2013; Good et al., 2021). The MBD plays a role in the binding ability of MeCP2 to methylated DNA, whereas the ID assists in the stabilization of the structure and binding capability of Methyl-CpG-Binding Domain (MBD). The TRD facilitates transcriptional repression through cooperation with the co-repressor components, and the CTD enables MeCP2-chromatin binding (Liyanage and Rastegar, 2014).

### 2.3 DNA binding of MeCP2 *via* the methyl-CpG-binding domain

**FIGURE 4:**
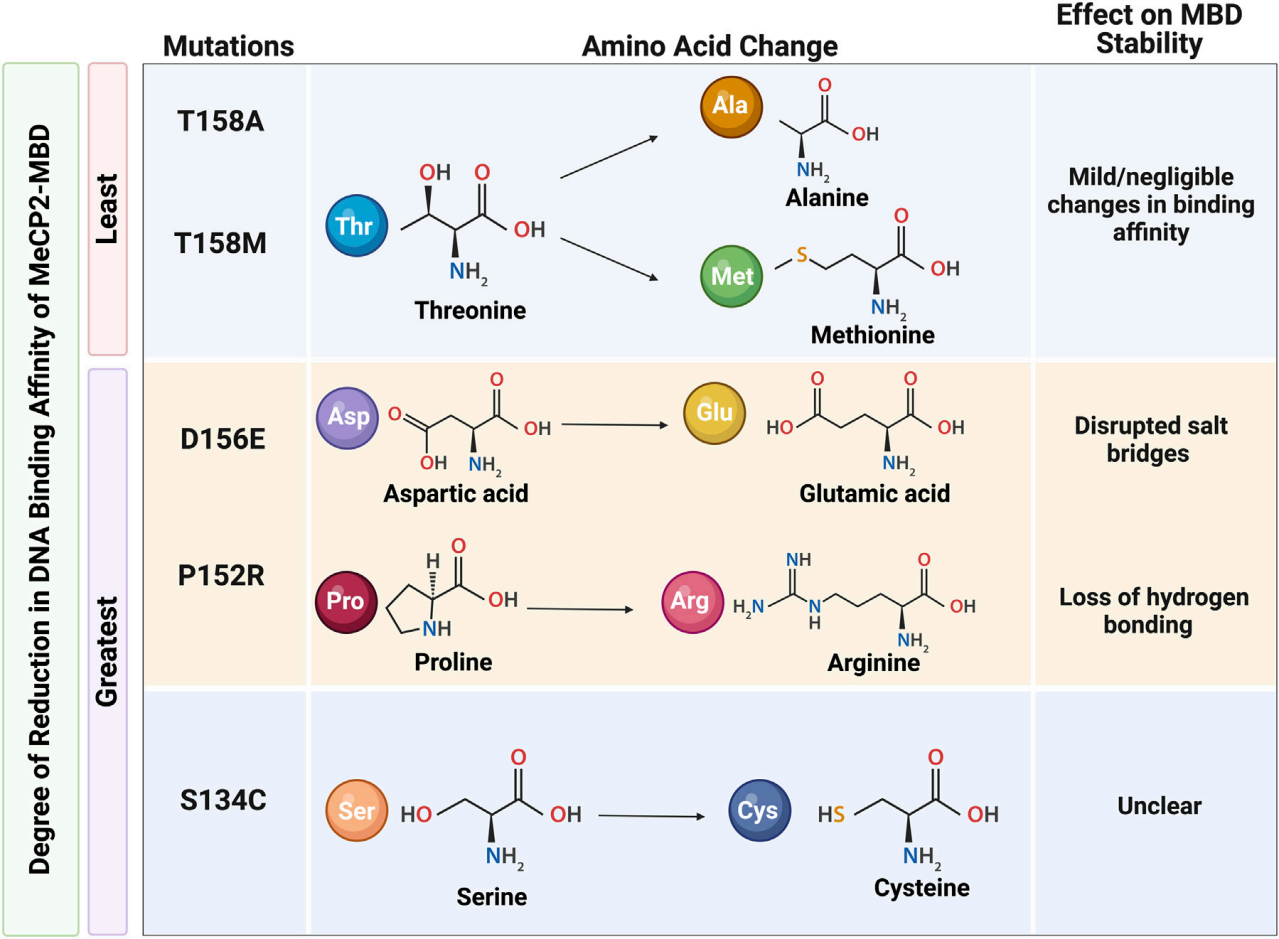
The effect on MeCP2 methyl binding domain (MBD) stability *due to* mutations affecting the DNA binding affinity of MeCP2. A) The mildest reductions in MBD binding affinity are observed in the T158A and T158M mutations; the greatest reductions in MBD binding affinity are observed in the S134C, P152R, and D156E mutations. Reduced DNA binding affinity in the P152R and D156E mutations are explained by loss of hydrogen bonding and disrupted salt bridges, respectively. However, the reason for great reductions in the MBD binding affinity associated with the S134C mutation remains unclear. Information for this Figure are extracted from Kucukkal et al., 2015 and Yang et al., 2016 (Kucukkal et al., 2015; Yang et al., 2016). Illustration created in BioRender.com.

Historically, MeCP2 has been shown to bind as a monomer to symmetrically methylated CpG dinucleotides *via* its methyl binding domain (Nan et al., 1996; Ghosh et al., 2010). However, later studies have challenged this notion as MeCP2 was found to exhibit cooperative dimeric binding to DNA (Ghosh et al., 2010; Khrapunov et al., 2014). Intriguingly, the full-length protein is not necessary for either recognition or binding to methylated CpG (mCpG) sites. Rather, the MBD (amino acid residues 78–162 based on MeCP2E2) is both necessary and sufficient for the binding of MeCP2 to mCpG (Nan et al., 1996). Meanwhile, MeCP2 protein domains flanking the MBD have been suggested to moderate the binding affinity of the protein to DNA (Claveria-Gimeno et al., 2017). The vitality of MBD to MeCP2-mCpG binding function has since influenced the structural characterization of the MBD as well as investigation into the effect of mutations on MeCP2 (Figure 4). Mutations in both the arginine-111 (Arg-111) and aspartate-121 (Asp-121) do not result in any detectable changes in the affinity of MBD for DNA, indicating the essential role of these amino acid residues in MeCP2 binding to mCpG. Contrastingly, mutations in other residues, such as arginine-133 (Arg-133), only mildly affect DNA binding. Further, research suggests that hydrophobic interactions between residues on the DNA binding surface (Tyr-123, Ile-125 and Ala-131) of MeCP2 contribute to the specificity of mCpG in the major groove of DNA (Free et al., 2001).

Moreover, RTT-causing *MECP2* mutations within the MBD affect MeCP2 DNA-binding affinity and may be clustered into three distinct categories based on the degree of effect on the DNA-binding affinity of the protein. The greatest reductions in binding affinity are found in the L110V, S134C, P152R, and D156E mutations. In contrast, a moderate increase in binding affinity is observed in the A140V and R111G mutations. Meanwhile, MeCP2 binding affinity is mostly unaffected or only slightly reduced in MeCP2 mutations with F155S, R106Q, R106W, R133C, and R133H. X-ray and nuclear magnetic resonance (NMR) analyses further elucidate the intramolecular consequences of these RTT-causing mutations. In accordance with the mild or negligible changes in binding affinity, neither the T158M nor the T158A mutations exhibit changes in the hydrogen bonding or salt bridges within the protein structure around the 158 amino acid residues, despite the difference in the pattern of hydrogen bonding between the wild type and mutant protein. On the other hand, reductions in DNA binding affinity in the case of D156E and P152R mutations may be explained by destabilizing nature of these mutations. While protein destabilization may be attributed to the loss of hydrogen bonding in P152R mutation, disrupted salt bridges may be responsible for reductions in DNA binding affinity of MeCP2 with D156E mutation (Yang et al., 2016). Interestingly, the S134C mutation has also been deemed as destabilizing to the MeCP2-MBD structure, yet this destabilization is not explained by disruptions in salt bridges or loss of hydrogen bonding (Kucukkal et al., 2015).

### 2.4 MeCP2, RNA splicing, and central nervous system

Alternative splicing of pre-mRNA is a complex process yielding a diverse pool of mRNA transcripts, which are translated into protein isoforms (Wang et al., 2015). Deregulation of alternative splicing has been implicated in neuronal development, neuronal signaling, and synaptic maturation. It is fitting, then, that aberrant splicing mechanisms underlie many neurological disorders. MeCP2-mediated alternative splicing has been linked to factors involved in synaptic organization and intracellular transport as well as chromatin organization and RNA processing (Cheng et al., 2017). As such, a variety of mechanisms have been proposed. For instance, it has been suggested that DNA methylation of alternatively spliced exons would promote MeCP2 binding and subsequent recruitments of histone deacetylases (HDAC) to influence the splicing mechanism of pre-mRNA (Maunakea et al., 2013). Apart from its interaction with methylated DNA to regulate RNA splicing, MeCP2 also interacts with several splicing factors. MeCP2 interacts with Y-box binding protein 1 (YB-1), an intrinsically disordered protein, which participates in pre-mRNA transcription and splicing, packaging of mRNA and regulation of mRNA stability, among other functions (Young et al., 2005; Good et al., 2021). Moreover, MeCP2 regulates splicing factors in alternative splicing events in mature neurons of the hippocampus and formation of the RNA-binding fox-1 (RBFOX)/(large assembly of splicing regulators) LASR complex splicing complex (Brito et al., 2020; Jiang et al., 2021).

Evidence from RTT mouse models have suggested a role for aberrant alternative splicing events in RTT pathology (Young et al., 2005). Rather than binding to mRNA itself, MeCP2 is assumed to control alternative splicing *via* interacting partners. This phenomenon is illustrated by the MeCP2-mediated regulation of the glutamate ionotropic receptor AMPA type subunit 2 (*Gria2/GRIA2*) gene. *Gria2/GRIA2* encodes the GluA2 subunit of tetrameric α-Amino-3-hydroxy-5-methyl-4-isoxazolepropionic acid (AMPA) receptors (Li, 2022). Generally, AMPA receptors mediate excitatory glutamatergic synaptic transmission. (Gan et al., 2015). One study in the cortex of *Mecp2* knockout mice found that both MeCP2 and lens epithelium-derived growth factor (LEDGF) are necessary for normal splicing of the *Gria2* exons (Li et al., 2016). The study also suggested that RTT-causing mutations within the MeCP2-TRD, in particular the R168X, R255X and R270X mutations, may impair the interaction between MeCP2 and LEDGF and impact the splicing of *Gria2* mRNA (Li et al., 2016). The effect of perturbed alternative splicing of mRNA of genes involved in synaptic transmission is suggested to explain the excitation/inhibition (E/I) imbalance observed in RTT patients and RTT mouse models. This E/I imbalance has been linked to impaired GABAergic and glutamatergic pathways in several brain regions including the hippocampus, cerebral cortex, amygdala, and brainstem (Li, 2022).

### 2.5 MeCP2 and chromatin architecture

MeCP2 is detected both at the euchromatic and heterochromatic compartments of the chromatin (Piccolo et al., 2019; Li et al., 2020). Through its interaction within the chromatin, MeCP2 regulates the accessibility and inaccessibility of transcription factors to chromatin, thereby regulating gene transcription. Further, the MeCP2-MDB interacts with the four nucleosomal histones (H2A, H2B, H3 and H4) with high affinity as well as linker histones (Ortega-Alarcon et al., 2024). The compacting ability of MeCP2 is suggested to be more potent that both histones H1 (mammalian linker histone) and H5 (avian linker histone), the known linker histones that are involved in binding the entry or exit sites of DNA on the nucleosomal core particle surface (Georgel et al., 2003; Hergeth and Schneider, 2015). Additionally, the pattern of nucleosomal array compaction differs between MeCP2 and linker histones. While linker histones create a zig-zag formation of decondensed nucleosomes, MeCP2 creates highly condensed ellipsoidal structures proposed to be formed by MeCP2-MeCP2 “bridges” or bivalent DNA-MeCP2-DNA “bridges” (Georgel et al., 2003). Consequently, MeCP2-compacted chromatin occupies about three times less volume as compared to H1/H5 linker histone-compacted chromatin. Furthermore, research proposes that the MBD itself is not sufficient to orchestrate chromatin condensation and MDB binding to methylated CpG sites is not considered a prerequisite for chromatin compaction (Georgel et al., 2003). While MeCP2 with R133C missense mutation retains its chromatin compaction properties despite its abolished recognition of methylated CpG sites, MeCP2 with nonsense R168X mutation is unable to form higher order chromatin structures, despite binding to the nucleosome arrays (Georgel et al., 2003). Thus, it is suggested that the chromatin compaction properties of MeCP2 reside in its TRD domain and/or CTD.

Furthermore, MeCP2 forms chromatin loops with undersaturated nucleosomal arrays *in vitro*. Undersaturated nucleosomal arrays comprised of about seven nucleosomes show no compaction. Instead, in the presence of MeCP2, free loops of DNA emerge from clusters of nucleosomes (Nikitina et al., 2007). The proposed mechanism of MeCP2-mediated chromatin loop formation involves a two-part process involving the binding of MeCP2 to methylated DNA followed by methylation-independent interaction between nucleosomes and MeCP2 CTD (Nikitina et al., 2007). Interestingly, studies reveal that MeCP2 mediates the silent chromatin-derived 11 kb chromatin loop at the Distal-less homeobox 5- Distal-less homeobox 6 (*Dlx5-Dlx6*) locus (Horike et al., 2005). Importantly, chromatin loop at the *Dlx5-Dlx6* locus was absent in chromatin from the brain of *Mecp2*-null mice. The identification of *Dlx5*/*DLX5*, a maternally expressed and imprinted gene, as a target of MeCP2 suggests a link between MeCP2 and genomic imprinting in RTT pathology. Moreover, studies indicated a possibility for MeCP2-dependent oligomeric chromatin “suprastructures” such as four-way binding junctions creating a structure similar to that of the “stem” motif (Georgel et al., 2003; Galvão and Thomas, 2005). However, the presence of this four-way junction *in vivo* has not yet been fully explored.

**FIGURE 5:**
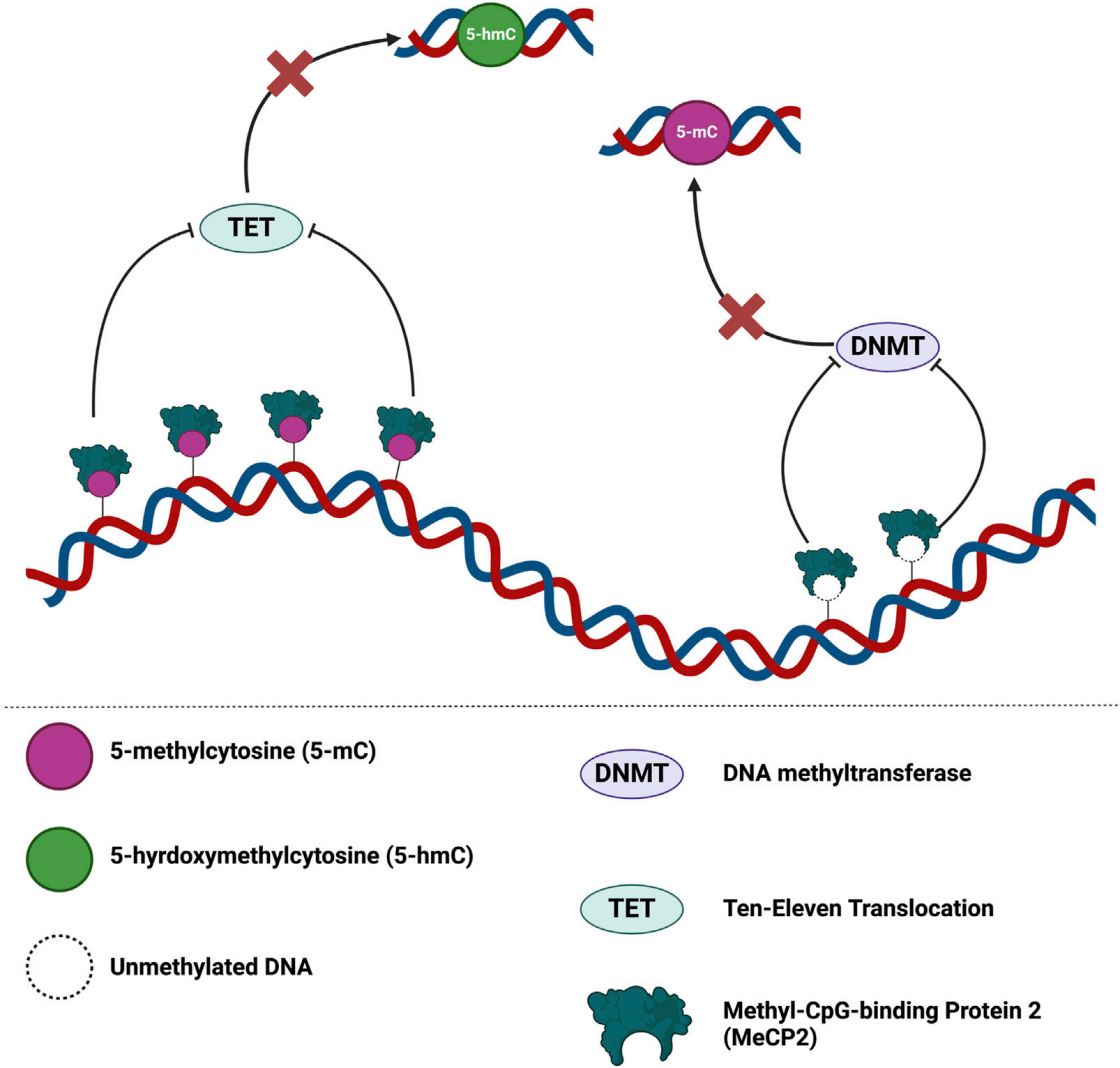
MeCP2 is a global guardian of the chromatin architecture and epigenome integrity. MeCP2 dosage may lead to different outcomes for epigenome integrity. Binding of MeCP2 to 5-mC may hinder TET activity and oxidation of 5-mC. At reduced levels of MeCP2, dysregulated modulation of the epigenome may permit promiscuous DNMT/TET activity at regions typically regulated by MeCP2 binding. On the contrary, increases levels of MeCP2 may disproportionately hinder DNMT/TET activity in the epigenome. Figure is adapted from Rastegar and Davie 2023 (Rastegar and Davie, 2023). Illustration created in BioRender.com.

MeCP2 also regulates chromatin architecture and globally impacts the epigenome by binding to unmethylated DNA *in vitro* (Figure 5). Binding of MeCP2 to unmethylated cytosine nucleotides prevents DNA methyltransferase (DNMT) writers and the subsequent conversion of cytosine to 5-methylcytosine (5-mC). Dysregulated MeCP2 binding to 5-mC may also hinder ten-eleven translocation (TET) protein activity and serial oxidation of 5-mC to 5-hydroxymethylcytosine (5-hmC), followed by 5-formylcytosine (5-fC), then 5-caroxylcytosine (5-caC) (Rastegar and Davie, 2023). Thus, this dysregulated binding of MeCP2 to unmethylated DNA, 5-mC, or 5-hmC may potentiate compromised DNA methylation events and/or DNMT/TET protein activities. These compromised DNA methylation events may then impact chromatin architecture and overall epigenome integrity (Rastegar and Davie, 2023).

### 2.6 MeCP2 and transcriptional regulation

Currently, MeCP2 has been recognized for its role in both transcriptional activation and repression *via* its interactions with various cofactors and its protein domains (Chahrour et al., 2008). Binding of MeCP2 to methylated CpG sites *via* its MBD in conjunction with interactions with multi-protein co-repressor complex involving histone deacetylases, HDAC1, HDAC2, and mSin3A at the MeCP2 TRD have been linked to repression of gene expression (Della Ragione et al., 2016). In addition to mSin3A, MeCP2 binds co-repressors N-CoR and c-Ski in MeCP2-mediatated transcriptional repression (Kokura et al., 2001). Moreover, MeCP2 recruits a host of chromatin-remodelling proteins to regulate transcription. For instance, MeCP2 associates with SWI/SNF complex, a catalyst for chromatin reorganization, in methylated cancer genes. These genes include *ATP Binding Cassette Subfamily B Member 1* (*ABCB1*) and *Thrombospondin-1* (*THBS1*), as well as the *fragile X messenger ribonucleoprotein 1* (*FMR1*) gene in Fragile X Syndrome to induce transcriptional repression (Harikrishnan et al., 2005). Furthermore, the MeCP2 N-terminal Domain (NTD) interacts with the chromo shadow domain of heterochromatin protein 1 (HP1) to modulate heterochromatin association during myogenic differentiation (Agarwal et al., 2007). Additionally, gene expression in neurons was shown to be transcriptionally regulated by the interaction between MeCP2 and the co-repressor for element-1-silencing transcription factor (Co-REST) complex followed by recruitment of the histone methyltransferase SUV39H1 (suppressor of variegation 3-9 homolog 1) and mSin3A (Ballas et al., 2005). Essentially, the MeCP2-HP1 interaction promotes association of HP1 with pericentric heterochromatin (Agarwal et al., 2007).

Historical models of MeCP2-mediated transcriptional regulation proposed that MeCP2 binds methylated CpG regions in gene promoters and recruits co-repressors to silence gene expression (Jones et al., 1998; Nan et al., 1998). However, one study suggests that MeCP2 promoter occupancy is not consistently associated with transcriptional silencing. In fact, certain gene protomers bound by MeCP2 appear to be transcriptionally active. For instance, while MeCP2 mediates *Brain Derived Neurotrophic Factor (BDNF)* silencing by binding to the promoter, MeCP2 binding at the *JUNB*/*RNASEH2A* locus was linked to both a reduction of *JUNB* transcript levels and an increase in *RNASEH2A* transcript levels (Yasui et al., 2007).

### 2.7 Target genes of MeCP2

#### 2.7.1 Neuronal targets of MeCP2

MeCP2 is about five-times more abundant in primary culture of cortical neurons than in glial cells—a testament to their crucial role in neuronal function (Zachariah et al., 2012). Within neurons, the absence of MeCP2 is involved in the direct and indirect regulation of several genes, many of which have increased expression (Urdinguio et al., 2008). MeCP2 binds to the promoter or other regulatory regions of several neuronal genes such as m*yelin-associated oligodendrocytic basic protein* (*Mob*), *FK506 binding protein 5* (*Fkbp5*), *Dopa decarboxylase* (*Ddc*), *Eyes absent 2 homolog* (*Eya2*), *Pleiomorphic adenoma gene-like 1* (*Plagl1*), *and S100 calcium binding protein A9 (calgranulin B)* (*S100a9*) and upregulate these genes in the midbrain, cortex and/or cerebellum (Urdinguio et al., 2008). Additionally, it is suggested that MeCP2 also transcriptionally regulates *interleukin-1 receptor-associated kinase 1* (*Irak1*), *delta-like 1 homolog* (*Dlk1*) and *proline dehydrogenase* (*Prodh*) albeit *via* an indirect mechanism (Urdinguio et al., 2008).

Another well-established neuronal target of MeCP2 is the *BDNF*/*Bdnf* gene. BDNF is known for its multifaceted role in neuronal maturation and synaptic plasticity (Binder and Scharfman, 2004) and is known to be regulated by MeCP2 (Abuhatzira et al., 2007). Specifically, MeCP2 has been shown to bind to the promotor III of *BDNF* in humans, and promoter IV of *Bdnf* in mice and represses its expression. (Chen et al., 2003; Cao et al., 2022). The vitality of MeCP2 regulation of *Bdnf* is underscored by the development of therapeutic strategies which attempt to restore BDNF levels in *Mecp2*-deficient mice as well as overexpression of BDNF in postnatal excitatory forebrain neurons of *Mecp2* knock out mice (Chang et al., 2006). Further, post-mortem studies in the human RTT brain indicate an impairment of the *MeCP2E1*/*E2*-*BDNF*-*miR132* homeostasis regulation (Pejhan et al., 2020). Together, these studies highlight a crucial role for MeCP2 in the regulation of *BDNF*/*Bdnf* in both RTT patients as well as RTT mouse models.

#### 2.7.2 Ribosomal targets of MeCP2 in neurons and RTT brains

The ribosomal targets of MeCP2 in the human brain are an emerging subject of research in the context of Rett Syndrome as mutations in the *MECP2* gene lead to various molecular and cellular alterations, including disruptions in ribosomal RNA (*rRNA*) transcription and maturation, which are mainly controlled by signaling by nucleolin and the pathways of mechanistic target of rapamycin (mTOR)–P70S6K (Olson et al., 2018). Deregulation of genes and proteins involved in *rRNA* processing and ribosome biogenesis have been also observed in fibroblast cells from RTT patients, potentially affecting general protein translation *due to* a reduction in mTORC1 activity (Pascual-Alonso et al., 2023). However, the interaction between MeCP2 and proteins involved in *rRNA* processing and mRNA splicing is still not fully understood (Pascual-Alonso et al., 2023).

Global impairment of RNA transcription and translation observed in human embryonic stem cells (ESCs) with *MECP2* loss-of-function in differentiated neurons further supports the relationship between MeCP2 and ribosomal targets (Li et al., 2013). In fact, a major group of genes affected in these *MECP2*-deficient neurons were those encoding ribosomal proteins (Li et al., 2013). Additionally, at two and four weeks of neuronal differentiation, these *MECP2*-deficient cells have nearly 30% and 50% less total RNA (respectively) as compared to their isogenic controls (Li et al., 2013). Collectively, these results suggest that MeCP2 loss-of-function mutations may affect protein synthesis in neurons and may account for impacted ribosomal targets and protein translation processes in the human RTT neurons. The impact of MeCP2 on its ribosomal targets in the human brain, particularly in the context of RTT, involves disruptions in *rRNA* processing, ribosome biogenesis, and/or protein translation processes (Olson et al., 2018; Buist et al., 2022; Pascual-Alonso et al., 2023). However, further research is needed to fully understand the specific interactions and mechanisms underlying MeCP2 dysfunction and its impact on ribosomal targets in Rett Syndrome. This understanding is essential for the development of targeted therapeutic interventions for this complex neurological disorder.

#### 2.7.3 Targets genes of MeCP2 in astrocytes, microglia and oligodendrocytes

While MeCP2 is vital for neuronal function, studies also suggest a role for MeCP2 in other brain cell types, including astrocytes. In one study utilizing mice, MeCP2E1 isoform levels were five times higher in primary neurons than in primary astrocytes (Zachariah et al., 2012). Despite this difference, loss of MeCP2 in astrocytes is suggested to be relevant to RTT pathology (Maezawa et al., 2009; Skene et al., 2010). Of genes identified to be dysregulated following the loss of MeCP2 in astrocytes, nine genes validated by quantitative real time-polymerase chain reaction (qRT-PCR) have been shown to be in line with pathways impacted by RTT (Yasui et al., 2013). These include *Apolipoprotein C-II* (*Apoc2*), *Cell adhesion molecule-related/downregulated by oncogenesis* (*Cdon*), *Cysteine and glycine-rich protein* (*Csrp*), *Iroquois related homeobox 3* (*Irx3*), *Leucyl/cystinyl aminopeptidase* (*Lnpep*), *Necdin* (*Ndn*), *Neuronal regeneration-related protein* (*Nrep*), *Solute carrier family member 38, member 1* (*Scl38 al*), and *Zinc finger, MIZ-type containing 1* (*Zmiz1*) (Yasui et al., 2013).

Research studies have also suggested a role for MeCP2 in microglial regulation. Particularly, it was found that microglia originating from *Mecp2*-null mice exhibited an impaired phagocytic capacity and response to immunological stimuli (Derecki et al., 2012). Cultured *Mecp2*-null microglia have been shown to release toxic levels of glutamate (an excitatory neurotransmitter), which damages dendrites and synapses (Maezawa and Jin, 2010). In fact, this neurotoxic glutamate release in *Mecp2*-deficient microglia has been linked to aberrant overexpression of the sodium-coupled neutral amino acid transporter 1 (SNAT1) glutamate transporter, which is encoded by *SLC38A1 solute carrier family 38 member 1* (*SLC38A*) gene, a target gene of MeCP2. In addition, one study suggests that MeCP2 binds to the promoters of *myelin basic protein* (*MBP*), *proteolipid protein* (*PLP*), and *BDNF* genes in oligodendrocytes (Sharma et al., 2018).

### 2.8 The epigenetics of Rett Syndrome

#### 2.8.1 The methylation status of neuronal genes

Several groups have documented the genome-wide binding of MeCP2, especially at regions of high mCpG density (Skene et al., 2010; Baubec et al., 2013). Additionally, MeCP2 binds to non-CG methylation (mCH, H could represent A, C, or T) sites which are minimal in the human fetal brain, but more abundant in the adult brain, particularly in neurons (not glial cells) of the pre-frontal cortex (Lister et al., 2013; Chen et al., 2015). Intriguingly, MeCP2 was found in gene bodies with high levels of mCH (Chen et al., 2015). As post-mitotic neurons retain their mCH marks, postnatal mCH accumulation coincides with several processes occurring in the developing brain including synaptic pruning and synaptogenesis (Lister et al., 2013). Early in mammalian development, DNA methyltransferase 3A (DNMT3A) catalyzes the methylation of mCA in neurons—an epigenetic mark which may potentially remain stable for the extent of the neuron’s life. The mCA mark was shown to subsequently recruit MeCP2 for gene regulation as increased levels of mCA and MeCP2 coincided with higher gene repression (Stroud et al., 2017). One study suggests that binding of MeCP2 to mCA sites particularly within long genes (>20 kbp) is one mechanism for the repression of those genes. Notably, in neurons lacking functional MeCP2, these long genes are upregulated (Sugino et al., 2014). In the context of RTT pathology, 466 MeCP2-repressed genes with consistent upregulation in the amygdala, cerebellum, hippocampus, hypothalamus, and striatum of *Mecp2* knockout mice have been found to be exceptionally long, as well as rich in mCA sites (Gabel et al., 2015). The most profound effects of this epigenetic dysregulation occurs at nine-week as compared to four-week-old mice which are largely asymptomatic, correlating with symptom progression (Baker et al., 2013). In fact, there is an 11% increase in the total number of long genes aberrantly regulated by mutant MeCP2 from four-week to nine-week of age (Baker et al., 2013). This evident increase in dysregulation of long genes expression, especially at the time of brain development, offers insight into the mechanisms of neuronal dysfunction observed in RTT, as long genes tend to play a role in neuronal development (Lopes et al., 2021).

Alterations in genomic 5-hmC were also studied in knockout mouse models of RTT. Here, *Mecp2* dosage exhibited negative correlation with overall abundance of 5-hmC in the cerebellum (Szulwach et al., 2011). Further, there was about 20% increase in 5-hmC abundance and about 25% decrease in 5-hmC abundance in the loss of *Mecp2* and overexpression of *Mecp2*, respectively (Szulwach et al., 2011). Additionally, researchers observed increased intragenic 5-hmC in male *Mecp2*-knockout mouse model (Szulwach et al., 2011). Intriguingly, this increase was restricted to the gene bodies with no increase in 5-hmC upstream of the transcription start site (Szulwach et al., 2011). These findings suggest a gene-body specific role of MeCP2 in areas harboring 5-hmC.

Although *MECP2* mutations occur in the germline, the neurological and associated behavioural manifestation of these mutations (*MECP2*/*Mecp2*) occur at early postnatal stages (after 6–18 months of development in humans; between 4–6 weeks in male mice) (Chen et al., 2001; Guy et al., 2001). It has been shown that the increase in MeCP2 during postnatal development coincides with the frequency of mCH in neurons (Rasmi et al., 2023). These corresponding epigenetic signatures support the notion that MeCP2 plays a crucial role in the developing brain. Indeed, functional MeCP2 is necessary for proper formation and maintenance of neuronal networks in late stages of postnatal brain development as well as in the mature brain—biological events that are otherwise disrupted in the case of MeCP2 mutations (Nguyen et al., 2012). Research has shown that the loss of MeCP2 induced by Tamoxifen treatment during late juvenile and adult stages would result in overall brain shrinkage, as well as compaction of neuronal cell bodies (Nguyen et al., 2012). Similarly, induced loss of MeCP2 also resulted in a decrease in the numbers and length of basal and apical dendritic branches of CA1 pyramidal neurons of the hippocampus (Nguyen et al., 2012). Furthermore, the induced loss of MeCP2 during late juvenile and adult stages results in abnormal morphology of hippocampal CA1 astrocytes as well (Nguyen et al., 2012). Collectively, these studies emphasize the role of MeCP2 in the regulation of various biological processes in concert with epigenetic markers, especially at sensitive developmental periods.

#### 2.8.2 Aberrant histone modifications in Rett Syndrome pathology

Loss-of-function mutations of MeCP2 have been associated with increased histone acetylation in highly repressed long genes in the context of the RTT forebrain (Boxer et al., 2020). In a mouse model of the R306C RTT-causing mutation located in the CTD of MeCP2, increased H3K9, H4K12, and H3K27 histone acetylation was found at both the TSS and gene bodies of upregulated long genes (Boxer et al., 2020). Significant reductions in HDAC activity associated with the MeCP2-NCoR complex was also observed (Boxer et al., 2020). Together, the increased histone acetylation and decreased HDAC activity support the observation of upregulated long genes. In fact, the same research group found that functional MeCP2 transcriptionally inhibits highly methylated long genes through binding to its protein partner, NCoR, which is known to be a repressor. In *Mecp2* knockout mice, increased RNA Polymerase II (Pol II) binding at the transcription start sites and H3K27ac (indicators of transcriptional activation) are also found at the TSS of upregulated genes (Boxer et al., 2020).

#### 2.8.3 Chromatin architecture in the brain of Rett Syndrome patients and model systems

Studies suggest the presence of MeCP2 at near stoichiometric levels with linker histone H1 in nucleosomes of neuronal cells, as both proteins induce compaction of chromatin *in vivo* and *in vitro* (Ghosh et al., 2010; Ito-Ishida et al., 2018). In fact, MeCP2 and histone H1 are suggested to compete for nucleosome binding sites (Ghosh et al., 2010). Thus, it is fitting that, in the context of cells lacking MeCP2, there is a two-fold increase in histone H1 expression (Skene et al., 2010). This relationship between MeCP2 and histone H1 has been explored further in the context of mouse models of RTT which recapitulate loss-of-function genetic mutations as well as *Mecp2*-null mouse models. Particularly, it was found that while the amount of nuclear DNA was consistent in the CA1 hippocampal neurons of wild type and *Mecp2*-deficient neurons of female murine brain, there was a 20% increase in DAPI-rich regions associated with loss of MeCP2. This increase in DAPI-rich region of the DNA was complemented with 65% re-distribution of H4K20me3 into dense pericentromeric DAPI-rich regions along with an 11% increase in total nuclear levels of H4K20me3 (Linhoff et al., 2015). Furthermore, the observed changes in chromatin architecture appeared to be cell type-specific associated with MeCP2-deficiency. While the loss of MeCP2 resulted in similar changes in chromatin architecture in both CA1 hippocampal pyramidal neurons and hippocampal dentate granule cells, the compaction of chromatin and associated re-distribution of H4K20me3 was not observed in cerebellar granule cells (Linhoff et al., 2015).

Moreover, studies suggest that RTT-causing mutations of MeCP2 interfere with liquid-liquid phase separation (LLPS) during heterochromatin formation. LLPS refers to the formation of biomolecular condensates of biomolecules such as proteins and nucleic acids (Hyman et al., 2014). These membrane-less, liquid-like droplets aid in the coordination of vital biological processes including signal transduction, DNA repair and chromatin organization (Gibson et al., 2019; Levone et al., 2021; Ladbury et al., 2023). Intact MeCP2 induces the clustering of repressed heterochromatin into dynamic architectural compartments that have the potential of being fused overtime. Specifically, it was shown that self-oligomerization of MeCP2 is necessary for liquid-liquid phase separation *in vitro* and clustering of repressed heterochromatin *in vivo* (Brero et al., 2005; Li et al., 2020). However, mutations in MBD, TRD, and NID disrupt MeCP2-mediatated LLPS *in vitro* and *in vivo*. While missense MeCP2 mutations in the MBD disrupt chromatin compaction, missense MeCP2 mutations in the TRD disrupt cooperative MeCP2-MeCP2 interactions in neurons (Li et al., 2020; Wang et al., 2020). These, and many other investigations, underscore the multitude of processes which may be affected by RTT-causing mutations of MeCP2 and, in the broader spectrum, may underly the manifestation of clinical symptoms of RTT.

### 2.9 Rescue of aberrant Rett Syndrome epigenetics

Research indicates a potential for restoration of aberrant RTT epigenetics using a variety of techniques. The role of serotonin receptor 7, 5-HT7R, in neuro-physiological processes, including cognition, synaptic plasticity, emotion, and memory, has been well elucidated (Crispino et al., 2020). Several neurological disorders, including cognitive and mood disorders, exhibit altered 5-HT7R-mediated signaling. Additional evidence suggest involvement of 5-HT7R in RTT and Fragile X Syndrome, rare neurological disorders characterized by cognitive impairments (Lee et al., 2021). Given the wide variety of biological processes upon which 5-HT7R intersects, research into the therapeutic stimulation of 5-HT7R, and its potential effects, has established 5-HT7R as a new therapeutic target of neurological disorders.

One group demonstrated a rescue of histone acetylation patterns in the brain of symptomatic female heterozygous mice with loss-of-function *Mecp2* mutation through pharmacological stimulation of 5-HT7R. Two months following a 7-day treatment with the synthetic drug, LP-211, a selective agonist of 5-HT7R, rescue of various epigenetic aberrations was observed in the loss-of-function *Mecp2* mice. Particularly, LP-211 treatment normalized the increased HDAC1-mSin3a expression in the cortex; the same treatment normalized the reduction in histone H3 acetylation and HDAC3-NCoR levels of the same brain region (Napoletani et al., 2021). In the hippocampus, LP-211 normalized the hyperacetylation which inducing HDAC3 levels in RTT mice (Napoletani et al., 2021). Interestingly, the same group reported rescue in several RTT-like behavioural symptoms (cognitive and motor deficits and spatial memory) as well as mitochondrial dysfunction in mice with the LP-211 treatment (De Filippis et al., 2015; Valenti et al., 2017).

Further, another group reported a change in high levels of H3K9 and H4K16 acetylation observed *in vitro* and more than 50% increase in survival of male RTT mouse model following intraperitoneal administration of TAT-MeCP2 fusion constructs (Steinkellner et al., 2022). The researchers demonstrated localization of MeCP2E1 and MeCP2E2 fused to a TAT protein transduction domain in the nuclei of CNS cells. Here, therapeutic doses of the fusion proteins were reported to successfully cross the blood-brain-barrier to alleviate the RTT-like phenotype of the male mice (Steinkellner et al., 2022). It was particularly found that the TAT-MeCP2E2 fusion construct reversed H4K16 hyperacetylation *via* recruitment of HDAC1. However, the TAT-MeCP2E1 fusion construct appeared to rescue neuronal morphology as well as increase mouse lifespan to a greater extent than the TAT-MeCP2E2 fusion construct. This difference in the effect of E1 or E2 fusion construct is in line with MeCP2E1 being the predominant MeCP2 isoform in the CNS and is regarded as the most relevant MeCP2 isoform in RTT pathology (Olson et al., 2014; Yasui et al., 2014). The isoform-specific functional role of MeCP2E1 is underscored though its regulation of dendrite maturation and neuronal capacitance among other roles (Rastegar et al., 2009; Kaddoum et al., 2013; Djuric et al., 2015). Indeed, the MeCP2E1 and MeCP2E2 isoforms exhibit different expression profiles in both the developing and adult brain (Olson et al., 2014).

## 3 Prader Willi Syndrome

### 3.1 Etiology and clinical symptoms

Prader-Willi Syndrome (PWS, OMIM 176270) is a rare multi-systemic, genetic disorder characterized by errors in genomic imprinting leading to epigenetic silencing through DNA methylation (Butler et al., 2019b). PWS has an estimated incidence of 1:10,000 to 1:20,000 with failure to thrive and hypotonia in infancy, small hands and feet, and hypogonadism (Butler, 1990). Generally, PWS follows a weighted score-based diagnosis criteria with major, minor, and supportive categories as outlined in Table 1. Further, PWS-associated symptoms differ with age as infants and pre-adolescents display fewer symptoms than their older counterparts (Cassidy, 1984). While a total of five points are required for a PWS diagnosis for individuals under the age of three, with four points derived from the major category, eight points are required for a PWS diagnosis for individuals 3-years-old and older (Cassidy, 1984). However, molecular genetic testing is required for confirmation of diagnosis as PWS-associated symptoms may be subtle or non-specific (Holm et al., 1993).

**TABLE 1:** Clinical Score-Based Criteria for Diagnosis of Prader-Willi Syndrome adapted from Holm et al. (1993) and (Gunay-Aygun et al., 2001).

Criteria	Features/Symptoms	Points allocated
Major	Deletion of the 5q11–13 region on high resolution (>650 bands) or additional types of cytogenetic and/or molecular deregulation in the Prader-Willi chromosome region	1
	Neonatal and infantile central hypotonia	1
	Issues with feeding during infancy	1
	Rapid weight gain between 1–6 years of age with central obesity	1
	Characteristic facial features (almond eyes, small mouth with thin upper lip, down-turned corners of the mouth)	1
	Hypogonadism	1
	Hyperphagia	1
	Global developmental delay (<6 years)	1
Minor	Infantile lethargy	0.5
	Behaviour problems	0.5
	Sleep disorders	0.5
	Short height	0.5
	Hypopigmentation	0.5
	Eye abnormalities (esotropia, myopia)	0.5
	Speech impairments	0.5
Supportive	High pain threshold	
	Early adrenarche	
	Osteoporosis	
	Temperature instability	
	Scoliosis and/or kyphosis	

Furthermore, revised diagnostic criteria have been proposed to prompt genetic testing, based on age and certain features (Gunay-Aygun et al., 2001). For example, in infancy up to age two, hypotonia and feeding problems are major indicators, while excessive eating paired with global developmental delay is a major indicator between the ages of 6 and 12, and during adolescence (13+ years) hypogonadism (Gunay-Aygun et al., 2001).

### 3.2 Genetics of Prader-Willi Syndrome

**FIGURE 6:**
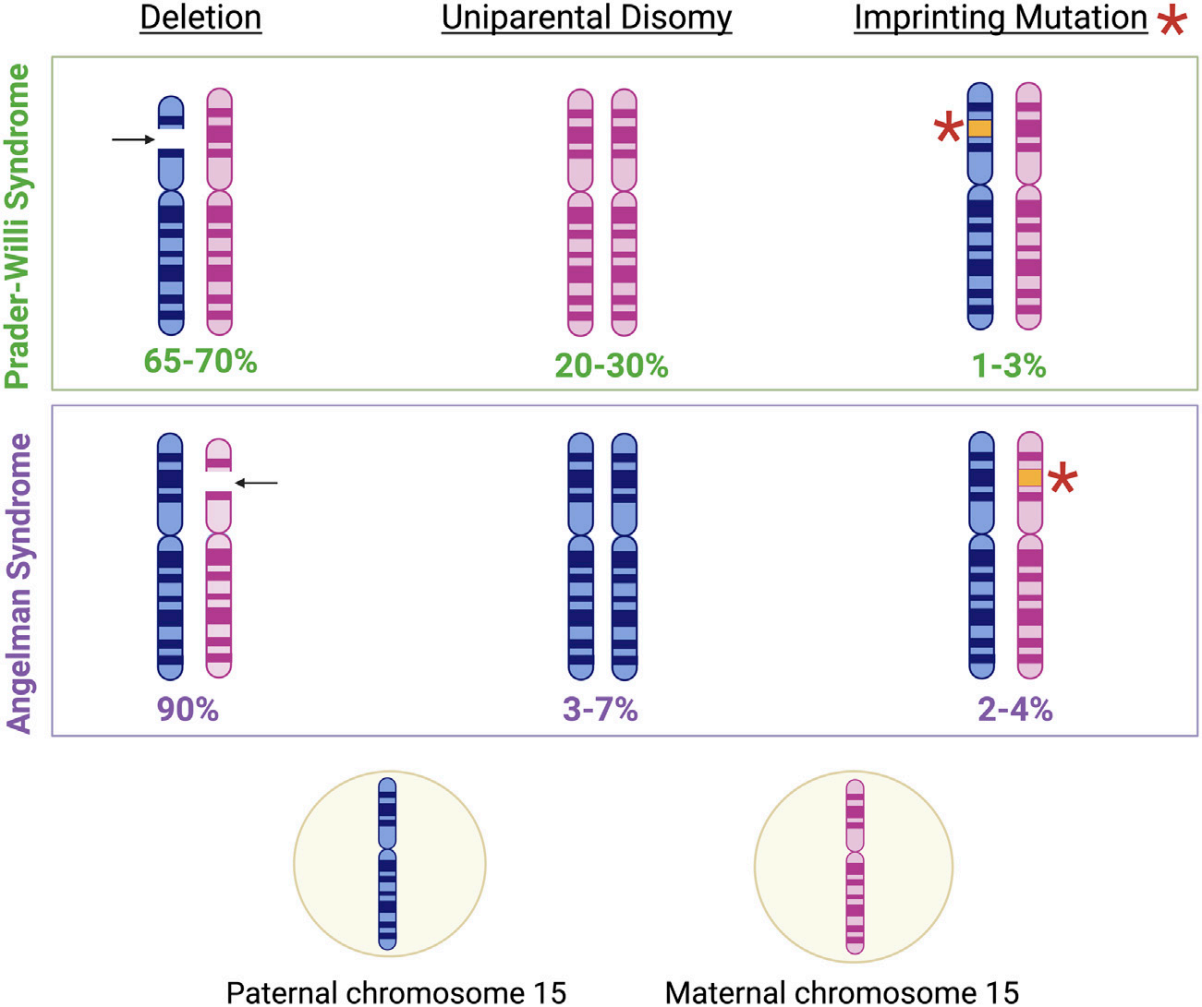
Genetic characteristics of Prader-Willi Syndrome (PWS) and Angelman Syndrome (AS). Approximately 65%–70% of PWS cases result from the deletion of genetic material in the paternal copy of chromosome 15, 20%–30% of PWS cases result from maternal disomy, and 1%–3% of PWS cases result from imprinting mutations in genomic regions associated with imprinting control on the paternal chromosome 15 (Cassidy et al., 2012). Meanwhile, approximately 90% of AS cases result from the deletion of genetic material in the maternal copy of chromosome 15, 3%–7% of AS cases result from paternal disomy, and 2%–4% of AS cases result from imprinting mutations in genomic regions associated with imprinting control on the maternal chromosome 15 (Dagli et al., 2012).

PWS is often classified as both a genetic and an epigenetic disorder linked to abnormal imprinting in the 15q11.2-q13.3 locus at the paternal *SNORD116* region (Holm et al., 1993; Cassidy et al., 2012). Specifically, PWS arises following the loss or absence of the expressed paternal copy of the *SNORD116* locus. While loss of the *SNORD116* locus majorly occurs by a large 6 mega-base deletion of the entire 15q11-q13 locus (60% of cases), maternal uniparental disomy (UPD) 15 (36% of cases), and imprinting defects (4% of cases) account for the remaining losses of the locus (Butler et al., 2019a). Additionally, loss of expression of *SNORD116* may also occur *due to* microdeletions (less than 1% of cases) in the upstream imprinting control region of *Small nuclear ribonucleoprotein polypeptide N* (*SNRPN*) causing the loss of the promotor (Duker et al., 2010). Intriguingly, loss of the same locus on the maternal allele results in Angelman Syndrome (AS), as discussed later (Figure 6) (Maranga et al., 2020).

### 3.3 Genes of the 15q11.2-q13.3 locus

The 15q11.2-q13.3 locus is divided into four distinct regions marked by three common deletion breakpoints: i) a proximal non-imprinted region, ii) the PWS paternal-only expressed region, iii) AS region comprised of maternally expressed genes, and iv) a distal nonimprinted region (Christian et al., 1995; Amos-Landgraf et al., 1999). Imprinted genes at the PWS/AS region are regulated by a bipartite *cis*-acting imprinting center (IC) which includes the PWS-IC and the AS-IC. Specifically, the PWS-IC regulates genes transcribed from the paternal allele while the AS-IC regulates genes transcribed from the maternal allele (Buiting, 2010; Mendiola and LaSalle, 2021).

### 3.4 Epigenetics of the Prader-Willi Syndrome imprinting center

The PWS-IC is regulated by a variety of parental allele-specific epigenetic alterations such as histone acetylation, histone methylation, and/or DNA methylation (Sutcliffe et al., 1994; Saitoh and Wada, 2000; Inoue et al., 2017). Currently, methylation of the CpG island of *SNRPN* is the most reported region of DNA methylation related to the PWS-IC. It is suggested that DNMT1 is responsible for imposition of DNA methylation in the PWS-IC as *Dnmt1-*deficiency results in the loss of the methylation in the PWS—IC as well as loss of the paternally expressed *Snrpn* (Li et al., 1992). The differential methylation of the PWS-IC imposed in the germline is subsequently maintained after fertilization. Furthermore, the entire region of 15q11-q13, and not only the PWS-IC, possesses continuous preferential paternal methylation as well as sparse “spikes” of maternal methylation. In fact, paternal differentially methylated regions (DMRs) are dispersed evenly with repeats and heterochromatin in the 15q11-q13 region. Meanwhile, maternal methylated DMRs are predominantly found in regulatory sites including promoters and transcription start sites (Kim et al., 2019).

Moreover, the paternally derived allele of *SNURF-SNRPN*, a well-studied imprinted gene locus of PWS, is correlated with acetylated histones as compared to its maternal counterpart which was found to be hypoacetylated (Saitoh and Wada, 2000). Specifically, paternal-specific histone H3 and H4 acetylation is observed in the *SNRPN* promoter region. However, maternal-based methylation of histone H3 on Lys9 and histone H4 on Lys20 as well as paternal-based methylation of histone H3 on Lys4 is observed in the *SNRPN* promoter region (Saitoh and Wada, 2000; Fulmer-Smentek and Francke, 2001; Xin et al., 2001). Further, analysis of the *NDN* promoter region revealed paternal-specific methylation at histone H3 on Lys4 (Xin et al., 2001; Lau et al., 2004). However, the purpose of this parental-specific histone methylation and acetylation pattern in the context of PWS has yet to be determined.

### 3.5 Epigenetic characteristics in Prader-Willi Syndrome diagnosis

Diagnostically, PWS is confirmed by abnormal DNA methylation within the PWS critical region which encompasses a variety of genes including *SNRPN*. In fact, this differential DNA methylation is the only technique that may be used to diagnose the three distinct genetic causes of PWS as well as distinguish PWS from Angelman Syndrome (Glenn et al., 1996; Glenn et al., 1997). Typically, the first exon of *SNRPN* as well as a transcription start site are located within a CpG island that is extensively methylated on the maternal allele, but unmethylated on the paternal allele (Kubota et al., 1996). Thus, the absence of the expressed paternal allele at *15q11-q13 due to* aberrant methylation as detected by Southern blot or methylation specific PCR analysis confirms a PWS diagnosis (Ramsden et al., 2010). Further, PWS diagnosis *due to* a deletion of *15q11-q13* may be confirmed by methylation sensitive multiplex ligation-dependent probe amplification analysis (MS-MLPA). Alternatively, if no deletion is observed, PWS may arise *due to* maternal uniparental disomy (UPD) or an imprinting defect and should be further validated by microsatellite analysis (Ramsden et al., 2010). While the underlying genetic cause of PWS may be determined using the aforementioned techniques, there are no particular phenotypes that may be associated with the genetic cause of PWS (Ramsden et al., 2010).

### 3.6 Epigenetic therapies for Prader-Willi Syndrome

Epigenetic therapies for PWS include the reversal of the silent PWS-ICR of the maternal allele. Early *in vitro* studies suggest that DNA methylation inhibitors, such as 5-Azacytidine and 5-aza-2′-deoxycytidine, cause the activation of *SNPRN* gene on the maternal chromosome (Saitoh and Wada, 2000; Fulmer-Smentek and Francke, 2001). One group also reported activation of the maternal copy of genes related to PWS using selective inhibitors of euchromatic histone lysine *N*-methyltransferase-2, UNC0638 and UNC0642. Use of these inhibitors resulted in the reduction of di- and tri-methylated H3K9 at the PWS-IC—histone modifications relevant for the *SNRPN* promoter region (Kim et al., 2017). Specifically, UNC0638 and UNC0642 inhibitors have been shown to reactivate *SNORD116* expression *in vivo* by reduction of histones at several loci within the PWS-IC (Kim et al., 2017). Reduction of tri-methylated H3K9 within the PWS-IC has also been achieved *via* inactivation of zinc finger protein 274, *ZNF274*, with a CRISPR/Cas9 technology, and results in reactivation of paternal *SNRP* and *SNORD116* clusters (Langouet et al., 2018). Furthermore, short hairpin RNA-mediated knockdown of the methyltransferase involved in tri-methylation of H3K9, *SETDB1*, facilitated the partial reactivation of *SNORD116* and other transcripts (Cruvinel et al., 2014). Together, these studies highlight possible targets of epigenetic therapy development for PWS.

## 4 Angelman Syndrome

### 4.1 Etiology and clinical symptoms

Angelman Syndrome (AS; OMIM 105830) is a rare neurological disorder affecting approximately 1:12,000 individuals. It is characterized by ataxia, microcephaly, speech limitations, intellectual deficits, muscular hypotonia with hyperreflexia, paroxysms of laughter, sleep disorders and seizures (Buiting, 2010; Buiting et al., 2016). Diagnosis of the disease typically occurs between one and four years of age with an onset of seizures in more than 80% of patients before the age of three (Margolis et al., 2015; Maranga et al., 2020). A combination of behavioural, clinical and developmental phenotypes, in addition to genetic testing, is used to diagnose AS (Bonello et al., 2017). However, AS is often misdiagnosed as other neurodevelopmental diseases, such as Mowat-Wilson syndrome, Rett Syndrome, Pitt-Hopkins syndrome, X-linked alpha-thalassemia syndrome, and SLC9A6 associated X-linked disorder, *due to* its similar clinical presentation (Bonello et al., 2017). The phenotypic similarity to other diseases, along with the subtly of signs at the onset of AS, is a major source of misdiagnosis and may contribute to underreported cases. Although patients with AS are expected to have a normal life expectancy, some patients die due to seizure complications (Bonello et al., 2017).

### 4.2 Genetics of Angelman Syndrome

Like PWS, Angelman Syndrome may arise from a variety of chromosome alterations at the 15q11.2-q13.3 locus including a 5–7 Mb *de novo* deletion, of chromosome 15 UPD or an imprinting defect (Buiting, 2010). Interestingly, the 5–7 Mb deletion in the 15q11.2-q13.3 locus has been linked to the most severe AS phenotype which involves seizures, microcephaly, motor impairments, and speech impairments (Valente et al., 2013; Keute et al., 2021). Generally, the absence of the expressed maternal allele at *15q11-q13 due to* aberrant methylation as detected by Southern blot or methylation-specific PCR analysis is what confirms an AS diagnosis (Ramsden et al., 2010). Also, the absence of the expressed maternal allele at *15q11-q13* by MS-MLPA confirms an AS diagnosis *due to* deletion of *15q11-q13*. Alternatively, if no deletion is observed, AS may arise *due to* paternal UPD or an imprinting defect and may be further validated by microsatellite analysis (Ramsden et al., 2010). Further, about 10% of AS cases arise *due to* mutations of the *E3A ubiquitin ligase* (*UBE3A*) gene, which encodes a protein involved in the ubiquitination protein degradation pathway as well as co-activation of steroid hormone receptors (Fang et al., 1999; Ramamoorthy and Nawaz, 2008). Mutations and deletions in the *UBE3A* gene account for the five molecular mechanisms which cause Angelman Syndrome. The five mechanisms include: Class I a large, *de novo* deletion of the maternal chromosome 15q11-q13; Class II, UPD of the paternal copy chromosome 15; Class III, abnormal methylation of maternal chromosome 15 allele; Class IV, mutations within the *UBE3A* gene; and Class V, clinical manifestation of AS phenotype despite the absence of chromosome 15 abnormalities (Lossie et al., 2001).

### 4.3 UBE3A, Angelman Syndrome, and epigenetic therapies

In humans, the *UBE3A* gene of the 15q11.2-q13.3 locus encodes three UBE3A protein isoforms formed *via* alternative splicing: UBE3A isoform 1, UBE3A isoform 2 and UBE3A isoform 3. Each isoform may be distinguished by their subcellular localization, overall abundance, and unique N-terminal sequence. Studies in hESCs and neurons suggest that UBE3A isoform 1 is the most abundant isoform and is located in both the cytoplasm and nucleus (Sirois et al., 2020). In the nucleus, UBE3A is found in the euchromatin-rich regions of the nucleus and is proposed to regulate the expression of genes (Burette et al., 2017). While full elucidation of the functions of UBE3A in the human body is yet to be fulfilled, UBE3A has been linked to functions in mitochondria (Panov et al., 2020), regulation of the glutamatergic synapse organizer, *Cbln1*, in excitatory neurons (Krishnan et al., 2017), and regulation of Golgi acidification (Condon et al., 2013). Interestingly, the UBE3A protein is detected in both pre- and post-synaptic neurons (Dindot et al., 2008). UBE3A has been detected in both excitatory and inhibitory neurons within the cerebral cortex as well as glial cells of the human brain, albeit to a lesser extent (Burette et al., 2017). Apart from its ligase and transcriptional co-activator functions, UBE3A is essential in early stages of neurodevelopment involving neuronal dendrite growth and maturation, and synaptic pruning (Valluy et al., 2015; Tonazzini et al., 2019; Furusawa et al., 2023; Rotaru et al., 2023).

Intriguingly, the loss of nuclear, but not cytoplasmic, UBE3A results in manifestation the of AS phenotype as evinced in both mouse models and human patients (Avagliano Trezza et al., 2019). To compensate for the loss of *UBE3A* function in Angelman Syndrome, research into the activation of the silent paternal copy of *UBE3A* in the brain has been explored. In neurons, the antisense transcript, *UBE3A-ATS*, silences the paternal *UBE3A* allele (Meng et al., 2012). Activation of silenced paternal *UBE3A* has been studied using different topoisomerase inhibitors, including etoposide, topotecan, irinotecan, and dexrazoxane (Huang et al., 2011). Specifically, *in vivo* administration of topotecan resulted in downregulation of *UBE3A-ATS* expression overlapping the paternal copy of *Ube3a* to reverse silencing. This effect of topotecan was observed in neurons of the cerebellum, hippocampus, neocortex, spinal cord and striatum (Huang et al., 2011). Additionally, reactivation of *Ube3a* was achieved using antisense oligonucleotides (ASOs), synthetic, single-stranded oligodeoxynucleotides with targeted binding to pre-mRNAs. Application of ASOs has led to rescue of behavioural and physiological abnormalities in mouse models of AS with *Ube3a*-deficiency (Milazzo et al., 2021). Moreover, human-targeted ASOs delivered to the CNS of cynomolgus macaques repressed *UBE3A-ATS* with subsequent reactivation of paternal *UBE3A* (Dindot et al., 2023). Results from studies such as these, have informed advancement of ASOs for treatment of Angelman Syndrome into clinical trials. In fact, phase I/II clinical trials have begun studying the safety and tolerability of various doses in the ASO, GTX-102, in pediatric participants diagnosed with AS (ClinicalTrials.gov, NCT04259281). Further, studies show that the inactivation of *Ube3a*-*ATS* by CRISPR/Cas9 editing at the level of DNA and RNA result in expression of sense *Ube3a* in the brain of Angelman syndrome mice by gene therapy approach with adeno-associated virus (AAV) to deliver Cas9 nuclease as well as guide RNA constructs (Wolter et al., 2020; Schmid et al., 2021). Another mechanism to achieve restoration of UBE3A in the brain explores the introduction of functional copies of *UBE3A*. Here, injected AAV vector carrying *Ube3a* in the hippocampus of *Ube3a*-deficient mice, established UBE3A in the hippocampus and improvement of only some behavioural phenotype (Daily et al., 2011). Moreover, introduction of hematopoietic stem and progenitor cells with a lentiviral vector that expressed *UBE3A* into mice rescued behavioural and cognitive impairments (Adhikari et al., 2021).

## 5 Rubinstein-Taybi Syndrome

### 5.1 Etiology and clinical symptoms

Rubinstein-Taybi Syndrome (RSTS; OMIM 180849) is a complicated rare autosomal disorder following a dominant pattern of inheritance that includes intellectual disability in addition to other symptoms with a frequency of one in every 100,000 births affected by RSTS (Roelfsema and Peters, 2007). Most of the RSTS cases are linked to mutations in the *CBP* (also known as *CREBBP* (*CREB-binding protein*)) (60% of cases) and *p300* (*EP300*) (E1A Binding Protein P300) (3% of cases) genes (Petrij et al., 1995; Roelfsema et al., 2005). Patients with RSTS have a specific facial appearance in addition to a variety of skeletal abnormalities, such as angulation, positioning, or phalanges and thumb halluces being duplicated that provide the broad thumbs and enormous toes that commonly lead to diagnoses. Neuroanatomical abnormalities, including agenesis of the corpus callosum, Dandy-Walker deformity, and more subtle anatomical issues, have occasionally been observed in the brains of RSTS patients (Mazzone et al., 1989; Milani et al., 2015). Research in psychology has demonstrated that RSTS patients have low IQs, reduced time periods of attention, and defective motor coordination (Schorry et al., 2008). While it is evident that developmental changes are the cause of several clinical signs of RSTS, research using animal models suggests that p300/CBP protein deficiency in the adult brain also plays a role in the cognitive decline associated with RSTS (Korzus et al., 2004).

### 5.2 The genetics of RSTS - *CREBBP* and *EP300*

Mouse models of RSTS exhibit traits resembling certain clinical presentations of this illness. As a result, the mutant mice serve as important experimental models for researching the syndrome’s genetic cause and assessing potential treatments. As previously mentioned, research on mouse mutants has demonstrated the functions that CBP and p300 play in the adult brain as well as during embryonic development, indicating that deficiencies at either stage are likely to have a role in intellectual disability in humans. Although the skeletal deformities in CBP and p300 hemizygous mice are in parallel to those reported in RSTS patients, their general brain anatomy is normal. *Cbp*-deficient mice have a more pronounced phenotype, with more severe facial dysmorphia, phalangeal alterations, asymmetry, scoliosis in the dorsal skeleton, and an imbalance or excess of ribs and vertebrae. These mutants have decreased size and weight as well, which is in line with RSTS’s characteristically stunted growth (Oike et al., 1999; Viosca et al., 2010).

CBP and p300 are lysine acetyltransferases (KATs) which mediate the acetylation of histones to regulate chromatin structure and gene expression (Ogryzko et al., 1996; Bedford and Brindle, 2012). Impaired neuronal histone acetylation is a crucial clinical signature that may be related to the manifestation of the symptoms, according to research on RSTS mouse models. Notably, studies conducted on lymphoblastoid cell lines derived from patients with RSTS have demonstrated comparable deficiencies in histone acetylation. Furthermore, the cognitive abnormalities shown in RSTS may potentially be attributed to aberrant CREB-dependent transcription. The finding that transgenic mice with brains producing a dominant negative variant of CBP and blood cell-lines from RSTS patients with mutations in the CBP lysine acetyltransferase domain show reduced CRE-dependent transcription lends credence to this notion. Moreover, increased CREB activity improves the impairments in synaptic plasticity in *Cbp* ^*+/−*^ mice (Alarcon et al., 2004). Studies using CBP heterozygous and forebrain-specific conditional knock out (cKO) mice suggest distinct activity-driven gene expression systems associated with CREB are impacted differentially by CBP loss or decrease. CBP deletion impacts the transcriptional program generated regarding environmental enrichment. However, this deletion only marginally influences the stimulation of immediate early genes in the hippocampus (Valor et al., 2013).

Furthermore, it is not apparent if p300 is required for transcription that is CREB-dependent (Roelfsema et al., 2005). While p300 can activate cAMP response element (CRE)-dependent transcription, studies using fibroblasts from *p300* ^*−/−*^ null mice treated with the catalytic subunit of protein kinase A (PKA) have demonstrated normal activation of a CRE-luciferase reporter. Additionally, CREB-dependent transcription was not specifically impaired in the hippocampus region of *p300* heterozygous mice according to gene expression profiling (Viosca et al., 2010).

### 5.3 The role of epigenetics in Rubinstein-Taybi Syndrome pathology

Histone acetyltransferase (HAT) activity is fundamental to both CBP and p300, which is a key epigenetic mechanism dysregulated in RSTS. Here, CBP and p300 lower the interaction between histones and DNA and neutralizing the positive charge by acetylating certain lysine residues on histone tails (Park et al., 2014; Van Gils et al., 2021). As a result, a relaxed chromatin structure forms, allowing transcription factors and RNA polymerase II to access the DNA for gene transcription (Park et al., 2014). Mutations in the HAT domain of CBP/p300 impair their ability to acetylate histones, resulting in a condensed chromatin state and transcriptional repression of target genes critical for development (Van Gils et al., 2021). Further, CBP and p300 act as transcriptional co-activators by interacting with various DNA-binding transcription factors. Mutations in the Kinase-inducible domain (KID) interacting domain (KIX) domain of CBP disrupt its binding to transcription factors like CREB, impairing transcriptional initiation (Park et al., 2014; Van Gils et al., 2021). Additionally, CBP and p300 acetylate and modulate the activity of transcription factors like p53, NF-κB, and others, affecting the expression of their target genes (Korzus, 2017; Van Gils et al., 2021).

CBP and p300 contribute in chromatin remodelling by recruiting ATP-dependent chromatin remodelling complexes like SWI/SNF to further open up chromatin structure and interacting with methyltransferases and demethylases to regulate histone methylation patterns that influence gene expression (Van Gils et al., 2021). Moreover, CBP and p300 participate in chromatin remodelling by recruiting ATP-dependent chromatin remodelling complexes like SWI/SNF to further open up chromatin structure and interacting with methyltransferases and demethylases to regulate histone methylation patterns that influence gene expression (Van Gils et al., 2021).

While not directly involved in DNA methylation, CBP and p300 interact with and regulate the activity of DNMTs, thereby influencing DNA methylation patterns and gene expression. The epigenetic dysregulation caused by CBP/p300 mutations in RSTS leads to widespread transcriptional changes, affecting the expression of numerous developmental genes, cell cycle regulators, and other key pathways, ultimately resulting in the diverse clinical features of the syndrome (Korzus, 2017; Van Gils et al., 2021). Targeting these epigenetic mechanisms with histone deacetylase inhibitors (HDACi) or other epigenetic modifiers has shown promise as a potential therapeutic strategy for RSTS by restoring normal gene expression patterns (Park et al., 2014).

## 6 Huntington’s disease

### 6.1 Etiology and clinical symptoms

With a prevalence of approximately 2.71–4.88 per 100,000 persons (Medina et al., 2022), Huntington’s disease (HD; OMIM 143100) is a rare genetic disorder which results in the progressive failure of the nervous system, causing severe motor, mental, and psychiatric symptoms. These include spontaneous movements, coordination difficulties, communication and swallowing defects, brain deterioration, and psychiatric troubles such as depression (Yanagisawa, 1992). Wheelchair-bounding is the progression of the disease and more severe cases result in complete immobility, along with mixed impediments (Harrington et al., 2014). Intellectual symptoms could occur sooner than a formal diagnosis and handling HD requires a proper effort between healthcare specialists and supporter networks to discuss its intricate difficulties and determine possible therapy for progressive consequences (Epping et al., 2013; Harrington et al., 2014). According to a study focusing on families with a history of HD, the standard age of onset of symptoms varied from 21 to 50 years (Wexler et al., 2004).

### 6.2 The genetics of Huntington’s disease

Gusella et al. (1983) recognized a relation between the HD gene and a polymorphic DNA marker on chromosome 4, revealing the location of 4p16.3. Subsequently, CAG repeat expansions within the *Huntingtin (HTT)* gene were shown to be responsible for encoding the Huntington protein, known as the main origin of HD. In contrast to healthy individuals with the CAG sequence repeats between 9 and 35 times and average median ranging from 17 to 20 repeats, HD patients usually have a CAG expansion that is over 35 repeats (Gusella et al., 1983). Similarly, there is an opposite relation between the expansion length and HD symptoms initiation age. Generally, the decline of age onset occurs following the increase of the expansion length (>35 repeats) (Saudou and Humbert, 2016).

### 6.3 DNA-protein/protein-protein interactions in Huntington’s disease

While the exact mechanisms underlying HD pathogenesis are complex and not fully understood, both DNA-protein and protein-protein interactions play crucial roles. For instance, mutant HTT (mHTT) can directly bind to DNA and influence gene transcription. It cooperates with transcription factors, co-activators, and co-repressors, changing the expression of several genes included in neuronal survival, metabolism, and other cellular processes (Irfan et al., 2022). Further**,** met ruins the DNA damage repair machinery and leads to the storage of DNA damage. This could originate from direct connections with DNA repair proteins or by intervening with their function indirectly through other pathways. In HD, mHTT tends to generate aggregates, which are poisonous to neurons (Irfan et al., 2022). Protein-protein interactions play a critical role in this aggregation. Several chaperone proteins, such as Hsp70 and Hsp40, cooperate with mHTT to control its folding and prevent aggregation. Nevertheless, when overwhelmed, these chaperones can also raise mHTT aggregation. Moreover**,** mHTT cooperates with several cellular proteins included in diverse cellular methods, containing vesicle trafficking, mitochondrial function, and synaptic transmission. Interruption of these protein-protein interactions influences neuronal dysfunction and deterioration in HD (Irfan et al., 2022). Recognizing the special DNA-protein and protein-protein interactions engaged in HD pathology is crucial for developing targeted therapeutic interventions. Research is in progress to explore these connections using procedures like chromatin immunoprecipitation sequencing (ChIP-seq). By revealing the molecular methods causing HD, scientists plan to recognize new drug targets and develop efficient therapies for this destructive disorder. The HTT protein function remains mainly obscure, but substantially affects different biological features (Schulte and Littleton, 2011).

### 6.4 Target genes of the Huntington protein

The Huntington protein, encoded by the *HTT* gene, is predominantly linked with HD. Although its precise role is not entirely comprehended, it is supposed to act in several cellular activities, involving intracellular transport, gene transcription, and apoptosis. Conversely, recognizing certain target genes of the Huntington protein has been a subject of continuing research (Makeeva et al., 2023). Some investigations indicate that mutant Huntington protein might dysregulate the expression of several genes included in different cellular purposes, causing the pathology of HD. These dysregulated genes could involve those contained in synaptic and mitochondrial function, oxidative stress response, and others (Makeeva et al., 2023). Determining precise target genes of the Huntington protein is intricate owing to its complicated roles and the complex molecular pathways engaged with HD pathology. Evolving research in this area and utilizing methods such as functional genomics, transcriptomics, and epigenomics reveal the special genes and pathways influenced by mutant Huntington protein (Makeeva et al., 2023).

### 6.5 Proposed epigenetic mechanisms of Huntington’s disease

Studies using cell lines and animal models have linked the neuropathology of HD to deficient neuronal histone acetylation and loss of CBP function. Therefore, it has been suggested that CBP is trapped in the aggregated protein clumps of mHTT present in patient brain tissues as well as in most disease-related experimental models. Cell toxicity and transcriptional dysregulation result from the sequestration of CBP, which depletes CBP in the nucleus. Nevertheless, not all experimental models of HD have been able to replicate this impact. Recent studies of soluble and aggregated mHtt indicate that soluble mHtt is more effective at lowering CBP levels than the aggregated form. The turnover rate of p300 is not changed by mHtt (Valor et al., 2013). This contrasts with the direct blocking of CBP KAT activity. This is another mechanism that impairs CBP enzymatic activity (Valor et al., 2013). The direct suppression of CBP’s KAT activity by mHtt can obstruct the functionality of CBP, highlighting the specific impact of mHtt on CBP as opposed to p300. These insights provide a better understanding of the distinct regulatory effects of mHtt on CBP and p300 (Steffan et al., 2001; Jiang et al., 2006; Giralt et al., 2012).

Modified DNA methylation patterns in the HD patient’s brain, particularly in *HTT* gene-related regions has also been observed. DNA methylation’s dysregulation might affect *HTT* gene expression, worsening the symptoms of the disease. Further, HD models have shown transformed histone modifications influencing to transforms of gene expression patterns and following disease progression (Hyeon et al., 2021). Moreover, dysregulation of miRNAs and lncRNAs has been associated with HD, influencing functions like neuroinflammation, synaptic function, and neuronal survival (Saba et al., 2022). It has also been suggested that epigenetic modifications can affect the transcriptional system, resulting in modified gene expression included in HD pathogenesis. Transcription factors, co-activators, and co-repressors may be influenced by epigenetic changes, aiding the dysregulated gene expression detected in HD (Hyeon et al., 2021).

## 7 Schinzel-Giedion Syndrome (SGS)

### 7.1 Etiology and clinical symptoms

Schinzel-Giedion syndrome (SGS; OMIM 269150) is a very rare congenital neurological disease caused by gain-of-function mutations in *SETBP1* gene (SET-binding protein 1/SEB/MRD29) and has only 50 reported cases worldwide (Duis and van Bon, 1993; Disorders, 2022; Zheng et al., 2024). SGS is classified into three distinct types (type I, complex and classical type; type II, middle type; type III, simple type), based on clinical features (Liu et al., 2018). Severe midface retraction, developmental delay, progressive atrophy of the brain, and frequent seizures are characteristics of this syndrome. SGS is a fatal disease with most SGS children dying before 2 years of age (Liu et al., 2018).

### 7.2 The genetics and inherent epigenetics of SGS

The *SETBP1* gene is found on chromosome 18q12.3 (Jansen et al., 2021). SETBP1, a transcription factor, has multiple functions: DNA replication, transcriptional regulation, suppression of PP2A activity by stabilizing SET proteins, and epigenetic regulation *via* its recruitment of KMT2A (an H3K4 methyltransferase) (Kohyanagi and Ohama, 2023; Whitlock et al., 2024). SETBP1 has an AT-hook DNA binding motif and is involved in the expression of homeobox proteins (HOXA9 and HOXA10). A hotspot (12 bp in exon 4) is present in the gene region coding for amino acids 868 to 871, known as the degron. This region of the SETPB1 protein regulates protein degradation. However, mutations (e.g., D874V) occurring outside of the degron may also affect SETBP1 protein stability (Zheng et al., 2024). *SETBP1* gene mutations result in a protein with increased stability, resulting in increased steady-state levels of SETBP1 and SET proteins (Antonyan and Ernst, 2022). SET is a reader of the unacetylated carboxy-terminal tail of p53 and is a negative regulator of p53 activity. SET prevents CBP/p300 acetylation at p53 target genes. SET is a component of the INHAT complex which inhibits the activity of lysine acetyltransferases. SET accumulation results in the improper usage of regulatory regions and enhancer-promoter interactions (Zaghi et al., 2023). SGS neural progenitor cells have low p53 activity resulting in increased proliferation and increased levels of DNA damage, resulting in neuron degeneration (Banfi et al., 2021).

One group investigated the role of SEPTBP1 as an “epigenetic hub” in relation to developmental genes (Piazza et al., 2018). For instance, the presence of three AT hook domains in SEPTBP1 suggest a role in DNA-binding. In fact, researchers found that SETBP1 binds to 277 genes. Further, upregulation of target genes by SEPTBP1 was observed at the transcriptional level, indicating a role for SEPTBP1 as an inducer of gene expression (Piazza et al., 2018). While the results of this study point towards an epigenetic mechanism underlying SGS pathogenesis, additional studies in different model systems may provide greater insight into the role of epigenetics in SGS.

## 8 Concluding remarks

The epigenetic mechanisms involved in rare neurological diseases are as variable and numerous as the impacted pathways and manifested phenotypes. Ironically, this heterogeneity in epigenetic mechanisms has allowed for the development of disease-specific diagnostic tools and continues to inspire therapeutic strategies. Undoubtedly, a thorough understanding of the epigenetic mechanisms, which contribute to the pathogenesis of rare neurological diseases is essential for the development of epigenetic-based therapies. In recent years, several groups have demonstrated the potential for the use of CRISPR/Cas9, pharmacological-based, and other methods of gene therapy in the treatment of rare neurological disorders with epigenetic components. In rare neurological diseases with genomic imprinting defects, attractive strategies have involved epigenomic editing to activate regions of silenced alleles as well as viral delivery of functional alleles. Despite the encouraging results of these studies, appropriate gene dosage remains a point of concern. The fine line between be therapeutic dosage and a dosage leading to a duplication syndrome must be carefully navigated. The epigenetic treatment of rare neurological diseases is further plagued by the challenges of the efficient delivery of CRISPR components as well as the possibility of off-target effects. Thus, thorough investigation of the mechanisms and efficiency of these developed therapies is required at both the pre-clinical and clinical stages. Nonetheless, therapeutic strategies for rare neurological diseases, such as those described in this literature review, offer promising options for treatment of rare neurological diseases for which there are no current cures.

Despite the similarities in clinical manifestation of the rare neurological diseases discussed in this review, the epigenetic mechanisms suggested to contribute to disease pathogenesis and/or progression distinguish one disease from another. Notably, Rett Syndrome has been linked to mutation of a DNA methylation reader, aberrant histone modification, and altered chromatin architecture as evinced in patients and model systems. Meanwhile, gene deletions and imprinting defects are featured in both Prader-Willi and Angelman Syndromes. Further, impaired HAT activity due to mutations in CBP/p300 accounts for the inability to remodel chromatin for transcriptional regulation of target genes critical for development as in the case of Rubinstein-Taybi Syndrome. Finally, modified DNA methylation patterns and mutations in DNA-binding proteins contribute to the etiology of Huntington’s disease and Schinzel-Giedion Syndrome, respectively. Overall, the complexity of the epigenetic contributions to rare neurological disease provides multiple therapeutic targets and remains a subject for further exploration.

## Figures and Tables

**Table:** 

**5-caC**	5-carboxylcytosine
**5-fC**	5-formylcytosine
**5-hmC**	5-hydroxymethylcytosine
**5-HT7R**	Serotonin receptor 7
**5-mC**	5-methylcytosine
***A2bp1***	*Ataxin 2 binding protein 1*
**AMPA**	α-Amino-3-hydroxy-5-methyl-4-isoxazolepropionic acid
***Apoc2***	*Apolipoprotein C-II*
**AS**	Angelman Syndrome
**ASO**	Antisense oligonucleotides
**AAV**	Adeno-associated virus
**CBP**	CREB-binding protein
***Cbp***	*CREB-binding protein*
***Cdon***	*Cell adhesion molecule-related/downregulated by oncogenesis*
**CNS**	Central Nervous System
**Co-REST**	Co-repressor for element-1-silencing transcription factor
**CRE**	cAMP response element
**CREB**	cAMP response element-binding protein
**CRISPR/Cas9**	Clustered Regularly Interspaced Short Palindromic Repeats/Cas9
***Csrp***	*Cysteine and glycine-rich protein*
**CTD**	C-terminal domain
***Ddc***	*Dopa decarboxylase*
***Dlk1***	*Delta-like 1 homolog*
***Dlx5***	*Distal-less homeobox 5*
***Dlx6***	*Distal-less homeobox 6*
**DMRs**	Differentially Methylated Regions
**DNMT**	DNA (cytosine-5)-methyltransferase
**DNMT1**	DNA methyltransferase 1
**DNMT3A**	DNA methyltransferase 3A
**E/I**	Excitation/Inhibition
**ESCs**	Embryonic stem cells
***Eya2***	*Eyes absent 2 homolog*
***Fkbp5***	*FK506 binding protein 5*
***Gamt***	*Guanidinoacetate methyltransferase*
***Gprin***	*G protein-regulated inducer of neurite outgrowth 1*
***Gria2***	*Glutamate ionotropic receptor AMPA type subunit 2*
**HD**	Huntington’s disease
**HDAC**	Histone deacetylases
***HTT***	*Huntingtin gene*
**IC**	Imprinting Center
**ICD**	International Classification of Diseases
***Irak1***	*Interleukin-1 receptor-associated kinase 1*
***Irx3***	*Iroquois related homeobox 3*
**K**	Lysine
**KAT**	Lysine acetyltransferase
**KIX**	Kinase-inducible domain (KID) interacting domain
**LLPS**	Liquid-liquid phase separation
***Lnpep***	*Leucyl/cystinyl aminopeptidase*
**LASR**	Large assembly of splicing regulators
**MBD**	Methyl-CpG-binding Domain
**MeCP2**	(protein), Methyl-CpG-binding protein 2
***MECP2***	*(human gene)*, *Methyl-CpG-Binding Protein 2*
***Mecp2***	(murine gene)*, Methyl-CpG-binding protein 2*
***Mef2c***	*Myocyte enhancer factor 2C*
***Mobp***	*Myelin-associated oligodendrocytic basic protein*
**MS-MLPA**	Methylation sensitive multiplex ligation-dependent probe amplification analysis
***MBP***	*Myelin basic protein*
***Ndn***	*Necdin*
**NTD**	N-terminal domain
**NMR**	Nuclear Magnetic Resonance
***Nrep***	*Neuronal regeneration-related protein*
***Oprk1***	*Opioid receptor kappa 1*
**p300**	E1A-associated cellular p300 transcriptional co-activator protein
**p53**	Tumor protein p53
**PKA**	Protein Kinase A
***Plagl1***	*Pleiomorphic adenoma gene-like 1*
***PLP***	*Proteolipid protein*
**PNS**	Peripheral Nervous System
***Prodh***	*Proline dehydrogenase*
**PWS**	Prader-Willi Syndrome
**qRT-PCR**	Quantitative real time-polymerase chain reaction
**Rbfox**	RNA-binding fox-1
***rRNA***	*Ribosomal RNA*
**RSTS**	Rubinstein-Taybi Syndrome
**RTT**	Rett Syndrome
***S100a9***	*S100 calcium binding protein A9 (calgranulin B)*
***Scl38al***	*Solute carrier family member 38, member 1*
**SETBP1**	SET-binding protein 1
**SGS**	Schinzel-Giedion syndrome
***SNAT1***	*Sodium-coupled Neutral Amino acid Transporter 1*
***SNORD116***	*Small Nucleolar RNA, C/D Box 116*
***SNRPN***	*Small Nuclear Ribonucleoprotein Polypeptide N*
***Sst***	*Somatostatin*
**TET**	Ten-eleven translocation
**TRD**	Transcriptional repression domain
**TSS**	Transcription start site
***UBE3A***	*Ubiquitin protein ligase E3A*
***UBE3A-ATS***	*Ubiquitin protein ligase E3A antisense transcript*
**UPD**	Uniparental disomy
**WHO**	World Health Organization
**Y**	Tyrosine
***YB-1***	*Y-box binding protein 1*
***Zmiz1***	*Zinc finger, MIZ-type containing*
***ZNF274***	*Zinc finger protein 274*
